# De Novo Design of High-Affinity HER2-Targeting Protein Minibinders

**DOI:** 10.3390/biom15111587

**Published:** 2025-11-12

**Authors:** Yize Zhao, Wenping Wei, Zijun Cheng, Min Yang, Yunjun Yan

**Affiliations:** Key Laboratory of Molecular Biophysics of the Ministry of Education, College of Life Science and Technology, Huazhong University of Science and Technology, Wuhan 430070, China; zhaoyize@hust.edu.cn (Y.Z.); weiwenping@hust.edu.cn (W.W.); m202372771@hust.edu.cn (Z.C.); ymyangmin@hust.edu.cn (M.Y.)

**Keywords:** protein de novo design, HER2, Minibinder, breast cancer

## Abstract

Human Epidermal Growth Factor Receptor 2 (HER2) is a key therapeutic target in breast cancer. However, the application of existing anti-HER2 antibody drugs is limited by such issues as large molecular weight and poor stability. In this study, a series of small protein minibinders targeting HER2 domain IV were de novo designed using the RFdiffusion method. Candidate molecules were selected through a combination of ProteinMPNN and AlphaFold2 screening, and their binding capabilities were further evaluated using *Escherichia coli* surface display coupled with flow cytometry analysis. By integrating molecular dynamics simulations, confocal fluorescence imaging, and isothermal titration calorimetry (ITC) experiments, a highly efficient minibinder (0_703_6) with nanomolar affinity and a smaller molecular size was finally identified. Compared with the existing drug molecules, the identified minibinder exhibited approximately threefold higher affinity and a threefold reduction in molecular size. This study provides strong support for the development of novel, stable, and easily expressible HER2-targeted therapeutic molecules and also offers new insights into the rapid development of robust breast cancer drugs that may serve as ideal alternatives to monoclonal antibodies.

## 1. Introduction

Human epidermal growth factor receptor 2 (HER2) is a transmembrane tyrosine kinase receptor that plays a critical role in the development and progression of various cancers, particularly breast and gastric cancers [1,2]. Recent studies have also documented HER2 overexpression in uterine cervical neuroendocrine carcinoma [2]. Elevated HER2 expression not only accelerates tumor progression but is also a well-established predictor of poor prognosis, largely due to its capacity to activate multiple downstream signaling networks [3]. In a retrospective study from a national reference laboratory, complete CEP17 deletions were rare (<0.01%). After integrated reviews using RAI1 as the control gene, 2/9 (22.2%) were classified as HER2-positive due to a HER2/RAI1 ratio of ≥2.0 [4]. Although monoclonal antibody-based therapeutics such as trastuzumab have revolutionized the management of HER2-positive cancers, the frequent occurrence of drug resistance and the intrinsic limitations in binding affinity and specificity underscore the pressing need for alternative therapeutic approaches [5].

Chimeric antigen receptor (CAR) T cell therapy has rapidly advanced as a next-generation immunotherapeutic strategy, showing substantial promise in clinical oncology [6]. The majority of current CAR designs utilize antibody-derived single-chain variable fragments (scFvs) to mediate antigen recognition [7]. Although scFvs are engineered by linking the variable domains of heavy (VH) and light (VL) chains, their conformational fragility often leads to improper folding or aggregation. These structural instabilities reduce the precision of target recognition and are a major factor contributing to CAR-T cell dysfunction [8,9]. Moreover, the relatively large size and limited affinity of scFvs further constrain their clinical applicability [10]. To address the above problems, Kim et al. previously performed a computational design of Rb-H2 (PDB ID: 6LBX) targeting domain IV of HER2; however, its large structural size and relatively weak affinity still pose challenges for clinical application in CAR therapies [11]. On the contrary, miniprotein minibinders, a novel class of protein ligands, are developed through computational design, characterized by high affinity and strong specificity toward defined targets [12]. Therefore, developing protein minibinders, which are smaller in size, possess high affinity, and exhibit structural stability to replace conventional scFvs, represents a potential direction for enhancing the efficacy of CAR therapies. Compared to conventional antibodies, these minibinders possess a more compact structure, enhanced stability, and reduced immunogenicity. With the aid of advanced computational tools, high-efficiency minibinders targeting virtually any protein of interest can be rapidly generated, making them a promising alternative to scFvs [13,14]. Recent reports show that minibinders distribute at higher levels within subcutaneous and intracranial tumors and more efficiently cross the blood–brain barrier [15]. In addition, orally designed IL-23R-directed minibinders outperform clinical antibodies in terms of GI stability, pharmacokinetics, and efficacy, with reduced production costs [14].

In recent years, deep learning and diffusion-based generative models, particularly the RoseTTAFold Diffusion (RFdiffusion) model, have significantly advanced the field of protein design [16]. Complementary pipelines like BinderCraft operationalize these advances by integrating epitope-guided design, rapid computational screening, and developability-oriented filtering [17]. Together, these approaches can not only generate entirely novel protein structures from random noise, but also refine existing backbones to enhance their targeting capabilities [18]. RFdiffusion has demonstrated outstanding performance in designing picomolar-affinity helical peptide minibinders, symmetric structural assemblies, and various minibinders targeting antigens such as GAPD and TNFR, highlighting its powerful structural generation capabilities and broad target adaptability [16,19,20]. Leveraging the advantages of this model, the present study aims to perform de novo design of small-sized minibinders targeting HER2, with the goal of precisely modulating the binding interface through RFdiffusion to achieve dual optimization of binding affinity and structural stability.

Therefore, in this study, the RFdiffusion technique is employed using the dl_binder_design pipeline to perform de novo design of high-affinity protein minibinders targeting HER2, using the trastuzumab epitope on domain IV of HER2 as the protein binding site [21]. The resulting minibinders are further validated at both the molecular and cellular levels for structural stability, specificity, and binding affinity. The findings of this study would provide valuable insights for the rapid development of therapeutics targeting HER2 and other clinically relevant membrane proteins.

## 2. Materials and Methods

### 2.1. Hydrophobicity Analysis of the Target Protein

The HER2 domain IV structure used in this section was derived from trastuzumab-bound conformation. After removing the antibody, we obtained the target structure for analysis by cleaning and trimming it according to the procedures described in the Methods and Supplementary Information. The design of the minibinder interface was informed by the spatial arrangement of hydrophobic residues on the target protein surface. To assess hydrophobicity, the HER2 domain IV sequence was subjected to analysis using ProtScale on the ExPASy Bioinformatics Resource Portal (https://web.expasy.org/protscale/ (accessed on 1 September 2024)) [22]. The Kyte and Doolittle hydropathy index was selected as the scoring scheme, with a window size of 9 and a linear weight model [23]. The resulting hydropathy score reflected the relative hydrophobicity of each region along the protein sequence: values greater than 0 indicate hydrophobic regions, and the higher the score, the stronger the hydrophobicity; scores below 0 correspond to hydrophilic regions. Three-dimensional surface mapping of hydrophobic residues on HER2 domain IV was conducted using PyMOL (v2.5, Schrödinger, LLC, New York, NY, USA). Highly hydrophobic patches on the target surface were selected as candidate interfaces for de novo minibinder design.

### 2.2. De Novo Design of Minibinder

The structure of the HER2 domain IV in its antibody-bound conformation (PDB: 1N8Z) was used as the template for minibinder design. Using PyMOL (v2.5, Schrödinger), the Repebody molecule, solvent molecules, and all heteroatoms were removed from the complex structure, and only the HER2 domain IV was retained. The structure was then cleaned and renumbered using the pdb_clean.py script provided by Rosetta to ensure standard formatting and continuous residue numbering. Structure-conditioned de novo design was performed using RFdiffusion, which applies a reverse diffusion process to generate backbone structures of putative minibinders around the defined hydrophobic interface of HER2 domain IV. Designed backbones were subsequently processed through the dl_binder_design pipeline, in which ProteinMPNN was employed for sequence design to optimize amino acid sequences compatible with the designed folds. The resulting minibinder–HER2 domain IV complexes, now containing both structural and sequence information, were further evaluated using initial guess AF2 to predict the complex structure and assess the binding interface. Full command lines (with all options), software versions and commit hashes, data portals/web interfaces, and exact parameter settings for each package are provided in the Supplementary Materials S2.

### 2.3. Molecular Dynamics Simulation and Binding Free Energy Analysis

Molecular dynamics (MD) simulations were performed using GROMACS version 2019.6 with the AMBER98 force field to investigate the structural stability and binding dynamics of the HER2 domain IV and its complex with the designed minibinder. The systems were immersed in a triclinic TIP3P water box, with neutralizing counterions added to balance the charge. Post-minimization, simulations underwent equilibration in NVT and NPT ensembles, and subsequently a 300 ns production run was executed under constant temperature (300 K) and pressure (1 atm), maintained through a velocity-rescaling thermostat and the Parrinello–Rahman barostat. For each system (HER2 domain IV alone and HER2–minibinder complex), two independent simulations were performed to ensure reproducibility. The root-mean-square deviation (RMSD) and root-mean-square fluctuation (RMSF) of the backbone atoms were calculated using built-in GROMACS tools to evaluate the dynamic stability of the structures over time. The binding behavior was visualized and analyzed using the last 50 frames (snapshots) from the final segment of the trajectory, which represented the equilibrated state of the complex. A representative conformation was selected as the medoid of the largest interface-RMSD cluster within the final segment of the trajectory (250–300 ns), as defined by the clustering analysis. To perform this, all conformations within the defined time window were grouped based on their interface-RMSD values, with the largest cluster corresponding to the most stable conformation. The medoid of this cluster was chosen as the representative conformation, which was the structure that minimized the distance to all other structures in the cluster, providing the most accurate representation of the binding mode of the complex.

### 2.4. Protein’s Expression and Purification

The codon-optimized nucleotide sequence encoding the extracellular domain IV of HER2 was chemically synthesized for expression in *Escherichia coli*. The gene fragment was subcloned via homologous recombination into a pET-28a expression vector (Tsingke Biotechnology Co., Ltd., Shanghai, China), containing a C-terminal GFP fusion and a 6×His tag for fluorescence visualization and affinity purification. For homologous recombination, the insert and the pET-28a backbone were prepared with 20–25 bp overlaps flanking the cloning site; the vector was PCR-linearized and treated with DpnI, and the purified fragments were assembled using the HieffClone One Step Cloning Kit (YEASEN, 10 911, Shanghai, China) at 50 °C for 30 min (vector:insert ≈ 1:3, 10 µL reaction). The assembly products were transformed into *E. coli* DH5a for selection on kanamycin plates, and positive clones were confirmed by colony PCR and Sanger sequencing prior to expression in BL21(DE3).

The recombinant plasmid was transformed into *E. coli* BL21 (DE3) cells. Protein expression was induced with 0.5 mM IPTG at the culture reached an OD600 of 0.6–0.8, followed by incubation at 18 °C for 20 h. Following harvest by centrifugation (5000 rpm, 10 min), the cells were resuspended in lysis buffer and disrupted by sonication. 1 mM PMSF was added to suppress protease activity. The cleared lysate was applied to a Ni-NTA affinity chromatography for protein purification. Non-specifically bound proteins were removed with 50 mM imidazole, and the target fusion protein (HER2 IV-GFP) was eluted with 250 mM imidazole. The eluted proteins were dialyzed at 4 °C for 2 h against 20 mM Tris buffer (100 mM NaCl, pH 8.0) to remove residual imidazole and salts.

The codon-optimized nucleotide sequence encoding the designed minibinder was chemically synthesized and cloned into the pET-28a vector with a C-terminal 6×His tag, followed by transformation into *E. coli* BL21 (DE3) competent cells. The procedures for recombinant protein production and purification were performed following the protocol described above. The purified minibinder was subsequently used for binding activity assays and structural characterization.

### 2.5. Flow Cytometry Analysis via Vesicle Adapted System

YiaT was used to anchor the minibinder on the surface of *E. coli*, enabling surface display [24]. Surface-displaying *E. coli* BL21(DE3) strains expressing minibinders were harvested, rinsed three times with PBS to eliminate remaining medium, and resuspended in PBS at the appropriate density. Resuspended bacteria were co-incubated overnight at 4 °C with 1 µM target GFP fusion protein under gentle rotation at ~20 rpm. After incubation, bacteria were washed three additional times with PBS and the final volume was adjusted to 1 mL of clean bacterial suspension for flow cytometry analysis. Flow cytometry was performed on a Beckman Coulter CytoFLEX flow cytometer using a vesicle-adapted protocol (vFC). A standard *E. coli* BL21(DE3) strain without minibinder expression was used to calibrate the instrument. Instrument settings were as follows: forward scatter (FSC) and side scatter (SSC) gain were set to 2500, and FITC (GFP) gains were set to 170. For each sample, data acquisition was carried out until 200,000 bacterial cell events had been collected. Flow cytometric results were subsequently processed with FlowJo software (v10.0, FlowJo, LLC, Ashland, OR, USA). A gating strategy was applied to exclude debris and select bacterial populations based on FSC and SSC parameters. Time gating was used to eliminate fluidic instability at the beginning of sample acquisition. Subsequently, a sequential gating hierarchy was applied: singlets were retained by excluding doublets on an FSC-A vs. FSC-H plot, and the final GFP^+^ gate in the FITC channel was defined from the BL21(DE3) negative control; the same fixed gates were then applied identically to all samples. GFP signal was detected through the FITC channel, and fluorescence-positive populations were defined using the negative control (*E. coli* BL21 without minibinder expression) to set the gating threshold. The mean fluorescence intensity (MFI) of each sample was calculated, and minibinder candidates with significantly elevated MFI compared to the control were selected for further analysis.

### 2.6. Circular Dichroism

Circular dichroism (CD) analysis was conducted on a JASCO J-1500 spectropolarimeter (JASCO, Tokyo, Japan). Proteins were dissolved in 30 mM potassium phosphate buffer (K_2_HPO_4_, pH 7.4) at a final concentration of 0.1–0.2 mg/mL. Far-UV circular dichroism spectra were collected in the range of 190–260 nm at 25 °C with a quartz cuvette of 1 mm optical path length. Samples were then rapidly heated to 95 °C, and the spectra in the same wavelength range were remeasured under denaturing conditions. After thermal denaturation, the samples were cooled and refolded at 25 °C for approximately 5 min before a third measurement was performed under refolding conditions. For thermal stability, ellipticity at 222 nm was recorded during a continuous temperature ramp from 25 °C to 95 °C at 1 °C/min; data were sampled every 5 °C after a brief equilibration (<30 s) at each set point.

### 2.7. Isothermal Titration Calorimetry (ITC)

Isothermal titration calorimetry (ITC) was performed on a MicroCal iTC200 system (Malvern Panalytical, Northampton, MA, USA) at 25 °C. The sample cell (200 μL) was loaded with the designed minibinder at a concentration of 0.013 mM, while the injection syringe (40 μL) was filled with HER2 domain IV at 0.156 mM. All proteins were prepared in 30 mM potassium phosphate buffer (pH 7.4) to ensure buffer consistency. A total of 20 injections were performed: the first injection was 0.4 μL (to minimize syringe backlash), followed by 19 injections of 2 μL each, with a 120 s interval and 4 s duration per injection. Stirring speed was set to 400 rpm throughout the experiment. Data were processed using Origin 7.0 (MicroCal version). Using a one-site binding model, the integrated heat data were fitted to obtain the thermodynamic parameters.

### 2.8. Cell Culture

The SK-BR-3 and MCF-7 cell lines were both purchased from the American Type Culture Collection (ATCC, Manassas, VA, USA). The specific culture conditions were as follows: SK-BR-3 cells: were cultured in RPMI-1640 medium supplemented with 10% fetal bovine serum (FBS), 100 U/mL penicillin, and 100 μg/mL streptomycin (purchased from Capricorn Scientific, Ebsdorfergrund, Germany); MCF-7 cells: were cultured in DMEM medium supplemented with 10% fetal bovine serum (FBS), and 100 U/mL penicillin and 100 μg/mL streptomycin (from the same source). All cells were routinely cultured in a constant-temperature incubator at 37 °C with 5% carbon dioxide (CO_2_) to maintain their normal growth status.

### 2.9. Confocal Microscopy

When SK-BR-3 and MCF-7 cells reached approximately 80% confluence, they were released from the culture surface using an enzyme-free buffer (Gibco, Thermo Fisher Scientific, Waltham, MA, USA) and inoculated at 1 × 10^5^ cells per well in glass-bottom 96-well coverglass plates (SPL Life Sciences Co., Ltd., Pocheon, Republic of Korea). Following a 24 h culture period in a CO_2_ regulated incubator (37 °C, humidified, 5% CO_2_), cells were treated with 300 nM GFP-tagged minibinder or GFP control protein for 3 h at 4 °C to promote membrane binding while minimizing endocytosis.

Following incubation, cells were gently washed three times with pre-warmed PBS to remove residual fluorescence and reduce background signals. Fluorescence images were acquired using an FV3000 confocal laser scanning microscope (Olympus, Tokyo, Japan) equipped with a 40× objective lens. All imaging parameters were kept consistent across experimental and control groups. Representative images were processed and analyzed using ImageJ software (version 1.53; National Institutes of Health, Bethesda, MD, USA).

## 3. Results

### 3.1. De Novo Design of Protein Minibinders Targeting on HER2 Domain IV

**Figure 1:**
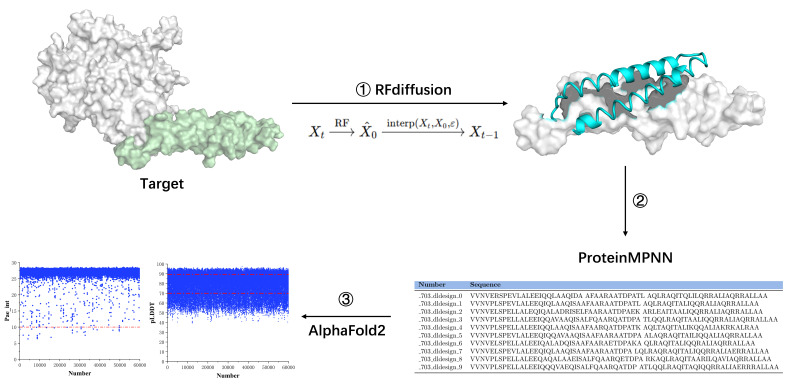
Overview of the computational workflow for de novo minibinder design. The minibinder design computational workflow process consists of three main steps. The target structure (HER2 domain IV) is preprocessed using PyMOL and Rosetta to remove non-essential regions and standardize the PDB format (step 1). RFDiffusion is used to generate initial minibinder backbone structures based solely on geometric constraints, without sequence information (step 2). ProteinMPNN(FastRelax) is applied to generate amino acid sequences fit to the designed backbones. The resulting minibinder–HER2 domain IV complexes are then evaluated using AlphaFold2, with pAE_interaction and pLDDT scores used for downstream filtering and selection (step 3).

Our objective was to develop a protein minibinder targeting domain IV of HER2, with the aim of either substituting traditional antibody fragments or serving as an alternative binding module for next-generation therapeutic and diagnostic applications. The minibinder was designed based on the surface hydrophobicity of the target protein to inhibit HER2-mediated cellular signaling or replace scFv as the chimeric antigen recognition domain in CAR T cell therapy, thereby achieving high specificity and high affinity. We employed RFdiffusion to perform diffusion-based generation targeting the HER2 protein, producing minibinder candidates with structural information but without sequence data. Subsequently, ProteinMPNN(FastRelax) was used to infer amino acid sequences based on the generated backbone structures. The resulting minibinders, now possessing both sequence and structural information, were complexed with HER2 domain IV and evaluated using AlphaFold2 to predict structures and score binding interactions, yielding the final selection results (Figure 1).

**Figure 2:**
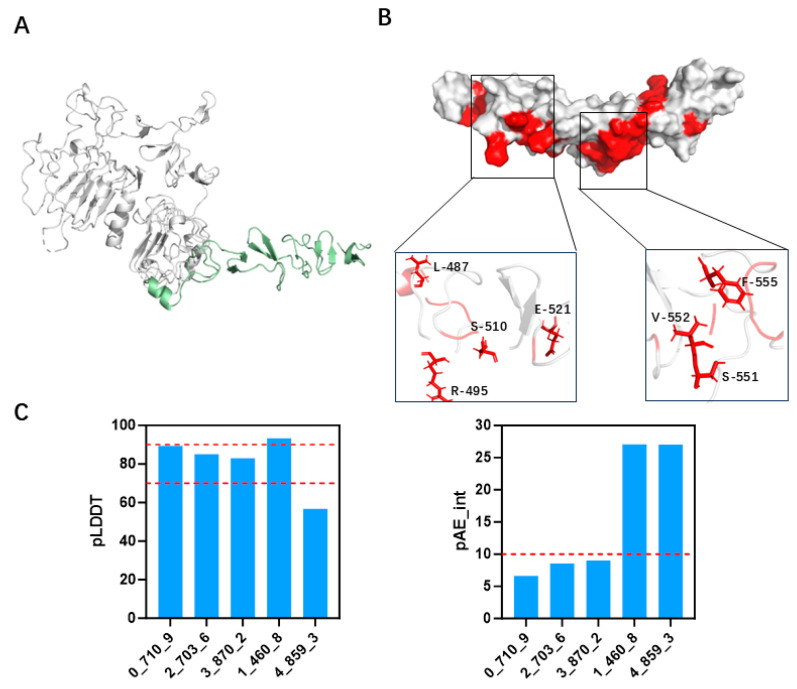
Hydrophobic surface analysis and minibinder design evaluation. (**A**) Processed HER2 structure highlighting domain IV, which is selected as the target region for minibinder design (shown in green). (**B**) Hydrophobicity analysis of HER2 domain IV, with hydrophobic residues are visualized in red, indicating potential interaction hotspots. (**C**) Evaluation of designed minibinders uses multiple screening criteria, including AlphaFold2-derived pAE_interaction and pLDDT scores.

To design a minibinder capable of specifically recognizing the target epitope, we first selected the structure of the target protein in its antibody-bound conformation as the design template. Specifically, the binding interface within the complex of the wild-type antibody (PDB ID: 3RFS) and HER2 domain IV (PDB ID: 1N8Z) was chosen as the structural basis for design. Using PyMOL, the antibody molecule as well as all solvent molecules and heteroatoms were removed (Figure 2A), retaining only the HER2 domain IV. The minibinder design was anchored to the trastuzumab epitope on HER2 domain IV and was guided by the hydrophobic characteristics of the target surface. A hydrophobicity analysis of HER2 domain IV was performed to identify surface-exposed hydrophobic residues, and regions with clustered hydrophobic amino acids were selected as candidate binding sites for design (Figure 2B and Figure S1). Minibinder scaffolds were generated using the RFdiffusion model, which produced a variety of glycine-based protein backbones with different topologies, including single-helix bundles, two-helix bundles, and three-helix bundles. A total of 5000 distinct scaffolds were obtained. Subsequently, amino acid sequences were designed using ProteinMPNN(FastRelax), and the structures of the designed minibinder–target complexes were predicted using AF2 initial guess along with initial binding pose estimations.

The obtained minibinder proteins predominantly exhibited two-helix and three-helix bundle architectures, or composite structures consisting of β-sheets and two-helix bundles. Most of the final minibinders adopted either two- or three-helix bundle topologies (Figure S2). Initial guess AF2 predictions were run on two-chain complexes comprising a truncated HER2 domain IV and each designed minibinder. The predicted aligned error for inter-chain distances (pAE_interaction) was used as an indicator of the confidence in the relative placement of the two chains in the predicted complex (lower values indicate higher confidence), whereas the predicted local distance difference test (pLDDT) score reflects per-residue local accuracy/model confidence. We applied screening thresholds of pAE_interaction < 10 and pLDDT > 70 based on AlphaFold2 predictions (Figure 2C and Figure S3). A total of 31 minibinder candidates met these criteria, most of which were composed primarily of α-helices (Table 1).

### 3.2. Screening and Specificity Assessment of Minibinders via Surface Display Technology

The DNA sequences encoding for the 31 minibinders sequences obtained in the previous step were chemically synthesized and subsequently fused with the gene encoding the *E. coli* surface display protein YiaT through homologous recombination (Figures S4 and S5). The resulting inserts were ligated into the pET-28a vector and transformed into *E. coli* BL21(DE3) for expression. Of these 31 candidates, 25 constructs were successfully cloned and yielded recombinant strains for minibinder expression, whereas six could not be recovered due to synthesis or cloning failures and were not pursued further (Table S1).

**Figure 3:**
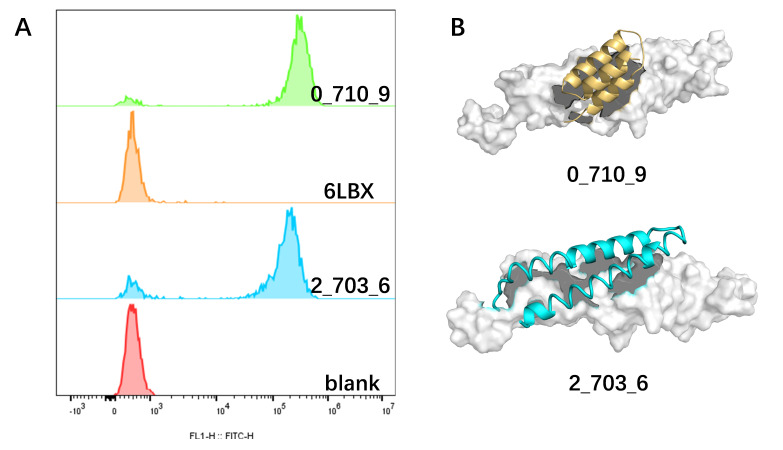
Flow cytometry analysis and structural modeling of designed minibinders. (**A**) Flow cytometry analysis of *E. coli* BL21 (DE3) displaying surface-displayed minibinders upon IPTG induction, following incubation with HER2–GFP. Each design was tested in three independent technical replicates (n = 3). (**B**) Predicted binding models of the selected minibinders in complex with HER2 domain IV. HER2 domain IV is shown in light gray, with minibinders colored in yellow and light blue, respectively. Structural models are visualized using PyMOL.

The designed minibinders, displayed on the surface of these strains, were co-incubated overnight with the fluorescently labeled target proteins. Among the 25 designed candidates, two minibinders, 0_710_9 and 2_703_6, exhibited prominent GFP fluorescence, indicating effective target recognition (Figure 3A). For the remaining designs, no detectable binding was observed; because surface-display levels were not quantified in this assay, we cannot exclude limited display as a contributing factor. As 6LXB was previously characterized by surface plasmon resonance (SPR) in a cell-free format, differences in construct design and assay context (GFP tagging, orientation/linker, and whole-cell co-incubation) may underlie this discrepancy; without quantitative display measurements, we cannot determine the relative contributions of expression versus assay-format effects. To further characterize the selected minibinders, structural modeling of the binding modes revealed that minibinder 0_710_9 adopted a three-helix bundle architecture, while 2_703_6 exhibited a two-helix bundle configuration (Figure 3B).

### 3.3. Binding Stability Differences Revealed by Molecular Dynamics Simulations

**Figure 4:**
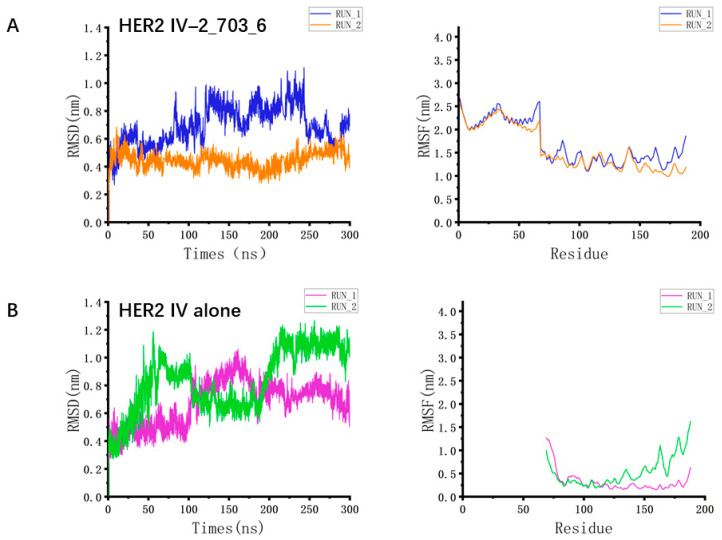
MD simulations of HER2 IV–minibinder complexes. (**A**) RMSD and RMSF profiles of the HER2 IV–2_703_6 complex over 300 ns. Residues 1–56 correspond to the minibinder, and residues 68–188 represent HER2 domain IV. (**B**) RMSD and RMSF profiles of HER2 IV alone over 300 ns, serving as a reference for structural fluctuations without minibinder association.

To evaluate the stability of the HER2 IV complexes with these two minibinders, molecular dynamics (MD) simulations were performed using the GROMACS (version 2022.2). The time evolution of the backbone RMSD of the minibinder–HER2 IV complexes revealed that 2_703_6 remained comparatively stable throughout the 300 ns simulation, indicating a robust and stable interaction. In contrast, 0_710_9 displayed larger-amplitude motions and did not converge to a single conformational basin within the 300 ns window in either replicate, which we interpret as a more dynamic binding mode under our simulation conditions rather than definitive instability (Figure 4A and Figure S6). The RMSF profiles of the minibinder–HER2 IV complexes derived from the MD trajectories showed that, although 0_710_9 exhibited slightly higher flexibility, the overall RMSF values were negligibly different differences between the two complexes. Notably, RMSD/RMSF primarily report on mobility; some flexibility can be compatible with—or even favor—binding depending on the mechanism. Moreover, the RMSF profiles of the HER2 IV domain in both complexes were higher than those of the unbound (free) HER2 IV. This is consistent with the intrinsically dynamic nature of HER2 domain IV, which can undergo subtle ligand-induced rearrangements; thus the higher RMSF likely reflects a redistribution of motions upon binding rather than global destabilization (Figure 4B).

Collectively, these results indicate that 2_703_6 establishes a stable interaction with HER2, closely resembling its designed conformation, whereas 0_710_9 may have limited capacity for sustained binding under physiological conditions.

### 3.4. Specific Binding of 2_703_6 to HER2 Confirmed by Cellular and In Vitro Assays

**Figure 5:**
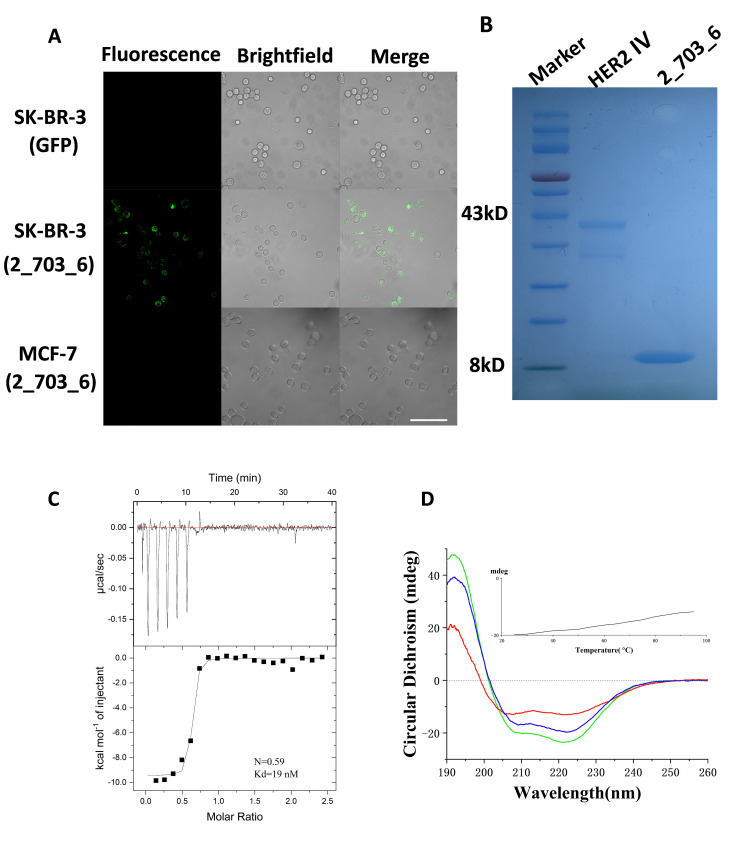
Binding of 2_703_6 to HER2-expressing cells and evaluation of their binding affinity and thermal stability. (**A**) Confocal fluorescence microscopy images of cancer cell lines treated with GFP-labeled 2_703_6. SK-BR-3 cells (middle), which overexpress HER2, and MCF-7 cells (bottom), which exhibit low HER2 expression, are incubated with 1 μM labeled 2_703_6 at 37 °C for 3 h. GFP alone serves as a negative control (top). Scale bar = 100 µm. (**B**) SDS–PAGE analysis of protein expression. Lane 1: molecular weight marker; Lane 2: purified HER2 domain IV (~43 kDa); Lane 3: minibinder candidate 2_703_6 (~8 kDa). (**C**) Binding affinity of 2_703_6 to HER2 is measured by ITC. (**D**) CD spectra of 2_703_6 recorded at different temperatures (green, 25 °C; red, 95 °C; blue, after cooling from 95 °C back to 25 °C). The inset shows CD signal at 222 nm as a function of temperature, indicating thermal unfolding and refolding behavior.

To investigate the binding behavior of the minibinder in a cellular context, the 2_703_6 sequence was fused with GFP via homologous recombination. The 2_703_6–GFP fusion protein was expressed, purified, and incubated with cancer cell lines of varying HER2 expression levels: SK-BR-3 (high HER2) and MCF-7 (low HER2). Confocal microscopy imaging revealed strong fluorescence at the periphery of SK-BR-3 cells, while only weak fluorescence was observed in MCF-7 cells. No fluorescence was detected in SK-BR-3 cells incubated with GFP alone (Figure 5A). These results indicate that 2_703_6 specifically binds to the extracellular domain of HER2 on the cell surface. However, cross-reactivity with domain IV of EGFR, HER3, or HER4 was not assessed in this assay; thus contributions from these receptors cannot be excluded. We further expressed the minibinder and HER2 IV–GFP in the *E. coli* BL21(DE3) expression system. Both proteins were purified by fast protein liquid chromatography (FPLC), and their purity was verified by SDS-PAGE (Figure 5B). The interaction affinity between the minibinder and HER2 was subsequently measured by isothermal titration calorimetry (ITC). Global fitting of the integrated heats to a one-site binding model yielded a stoichiometry parameter N = 0.59 and an affinity of Kd = 19 nM (Figure 5C). The sub-stoichiometric N value is consistent with a combination of factors, including a reduced active fraction of the recombinant proteins, partial oligomerization that decreases the number of accessible binding sites, and small assay-related concentration uncertainties. Compared with the trastuzumab Fab in PDB 1N8Z, 2_703_6 displays lower affinity but supports specific cell-surface labeling under our assay conditions. Next, to evaluate the thermostability of 2_703_6, we performed circular dichroism spectroscopy analysis. The circular dichroism spectrum of the purified minibinder displayed characteristic negative bands at 222 nm and 208 nm, along with a positive band at 193 nm, consistent with a typical α-helical bundle conformation. These spectral features are in good agreement with the predicted secondary structure from our computational design (Figure 3B and Figure 5D). As the temperature increased from 25 °C to 95 °C, the circular dichroism signal of the minibinder at 222 nm showed a slight enhancement, and more than half of the mean residue ellipticity remained at 95 °C, indicating that the melting temperature (Tm) of the minibinder exceeds 95 °C (Figure 5D, inset). Furthermore, after rapid refolding from 95 °C back to 25 °C, the CD spectrum showed no significant difference compared to the original spectrum at 25 °C, demonstrating that our design possesses excellent thermal stability (Figure 5D and Figure S7).

### 3.5. Structural Analysis of the 2_703_6–HER2 Domain IV Complex

**Figure 6:**
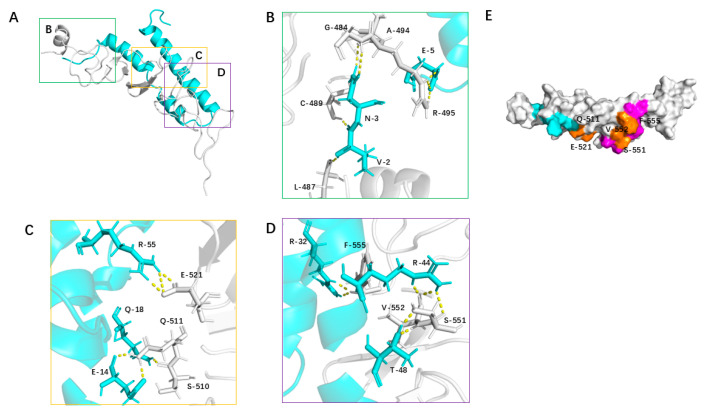
Structural analysis of the 2_703_6–HER2 domain IV complex. (**A**) Overall structure of the 2_703_6–HER2 domain IV complex. Both 2_703_6 and HER2 domain IV are shown as cartoon representations. Three key interaction regions are highlighted and detailed in panels (**B**–**D**). (**B**) Hydrogen bonds at the interface are shown as dashed lines in stick representation. (**C**) Hydrogen bonds (dashed lines) and hydrophobic interactions involving interface residues are shown in stick representation. (**D**) Both hydrogen bonds and salt bridges are depicted as dashed lines in stick representation, illustrating the electrostatic contributions to binding. (**E**) Comparison of the binding interfaces of 2_703_6 (light blue) and the previously reported binder 6LXB (magenta) on HER2 domain IV. Overlapping HER2 residues involved in binding are highlighted in yellow.

To visualize the binding mode of 2_703_6, we examined the equilibrated segment (250–300 ns) of the GROMACS trajectory. A representative conformation was defined as the medoid of the largest interface-RMSD cluster within this window (see Section 2), and all interaction occupancies were evaluated across the full trajectory. In this representative conformation, HER2 domain IV primarily engages the concave surface of 2_703_6 via hydrophobic contacts, hydrogen bonds, and salt bridges (Figure 6A). At the binding interface between 2_703_6 and HER2 domain IV, Glu5 of 2_703_6 forms a stable salt bridge with Arg495 of HER2. Additionally, Asn3 establishes hydrogen bonds with Gly484 and Cys489, while Glu5 also engages in contacts with Ala494. Moreover, Val2 forms hydrophobic contacts with Leu487, further contributing to the stabilization of the local binding conformation (Figure 6B). Glu14 of 2_703_6 forms multiple hydrogen bonds with Ser510 and Gln511 of HER2, while Gln18 also engages in polar interactions with Gln511, together establishing a stable hydrogen-bond network. Additionally, Arg55 forms a strong salt bridge with Glu521, providing substantial electrostatic stabilization to this region of the complex (Figure 6C). Thr48 of 2_703_6 forms dual hydrogen bonds with Ser551 and Val552 of HER2, while Arg44 also engages in polar interactions with Ser551. Notably, a cation–π interaction is observed between Arg32 and Phe555, further enhancing the binding affinity of the complex (Figure 6D). Subsequently, we compared the binding interface of 2_703_6 with that of the HER2-targeting minibinder designed by Tae Yoon Kim. The two interfaces share several residues, including Gln511, Glu521, Ser551, Val552, and Phe555 of HER2 (Figure 6E). These results indicate that the de novo–designed minibinder exhibits high specificity and strong affinity for HER2, highlighting its potential as a promising lead molecule for further development.

## 4. Discussion

Breast cancer is one of the most common malignant tumors among women in China, with the highest incidence and mortality rates [25]. Chemotherapy and endocrine therapies, including tamoxifen and doxorubicin, are widely used in the treatment of breast cancer [26]. However, the widespread use of these drugs has also led to challenges in treatment due to drug resistance, adverse side effects, and tumor heterogeneity [27,28,29]. In recent years, researchers have focused on developing monoclonal antibody drugs targeting key driver molecules or signaling pathways in breast cancer to achieve more precise targeted therapies [30,31]. Studies have confirmed that HER2-specific antibodies, such as trastuzumab, play a critical role in improving the survival rates of breast cancer patients [32]. Although antibody-based drugs are effective tools for blocking tumor cell signaling due to their high specificity and strong affinity, conventional antibodies still have certain limitations. For example, they have a large molecular weight (approximately 150 kDa), poor tissue penetration, and limited ability to access binding sites within solid tumors due to their low binding site density. In addition, their production is costly, development cycles are long, and they may induce immunogenic responses [33]. These factors have, to some extent, limited the widespread clinical application of antibody-based therapeutics.

In recent years, the field of de novo protein design has advanced significantly, providing an exciting opportunity to develop smaller, more efficient protein-based therapeutics, such as minibinders. Comprehensive reviews of cancer therapeutics—spanning modalities introduced before 1970, those developed from 1970 to 2023, and prospective future interventions—have explicitly linked molecular design strategies to the evolving treatment paradigm, underscoring that computational protein engineering is increasingly bridging the gap between discovery and application [34]. Compared to Rosetta-based approaches, the RFdiffusion method developed by Baker et al. indeed generates protein backbone models by fine-tuning the RosettaFold structure prediction network and has demonstrated superior performance in the design of protein minibinders, thus offering greater advantages [16,35]. In this study, we selected the highly conserved and specific structure of HER2 domain IV as the design target. The hydrophobic amino acids are dispersed and localized within the domain IV region (Figure 2B), making it suitable for minibinder design using the RFdiffusion method. The designed minibinders are composed of α-helical structures with molecular weights ranging from 6 kDa to 11 kDa, which are significantly smaller than monoclonal antibodies, consistent with previous reports [16,18].

The isothermal titration calorimetry (ITC) confirmed the binding between 2_703_6 and HER2 with an affinity of 19 nM, demonstrating strong binding potency sufficient to meet the clinical efficacy standards of therapeutic antibodies [36,37]. The stoichiometry parameter (n) was approximately 0.6. We consider that the sub-stoichiometric value of N primarily arises from several non-ideal factors. Both GFP–HER2 IV and 2_703_6 are recombinant proteins, and the fraction of properly folded, active species is likely lower than 100%. In addition, a portion of the proteins may undergo oligomerization, which would reduce the number of accessible binding sites and introduce additional heat effects. Finally, protein concentrations were determined using the BCA assay, which is susceptible to systematic errors due to differences between the sample buffer and the calibration standards. Together, these factors may lead to an underestimation of N and a slight deviation from the ideal 1:1 stoichiometry, while still remaining overall consistent with a one-site binding model. Amelie Eriksson Karlström et al. successfully designed a DARPin (minibinder) with a nanomolar-level binding affinity by developing a PNA-based pretargeting system via sortase A-mediated conjugation, which demonstrates its potential in immunodiagnosis for human therapy [38]. Cao et al. designed minibinders targeting the spike protein on the surface of SARS-CoV-2. These proteins can tightly bind to the viral spike and block its interaction with ACE2, thereby effectively preventing SARS-CoV-2 from infecting mammalian Vero E6 cells [33]. We also confirmed in cellular assays that the designed 2_703_6 bound well to the target in SK-BR-3 cells. Although 2_703_6 does not measurably block HER2 dimerization in our assay, domain-IV engagement may still attenuate signaling by biasing the ectodomain toward non-productive dimer conformations, limiting ectodomain shedding and generation of p95HER2, and/or promoting receptor internalization and down-regulation. These non-mutually exclusive mechanisms provide plausible routes for functional activity even in the absence of direct dimerization blockade. While functional inhibition remains to be tested, these data together suggest that 2_703_6 has promising potential for antitumor activity.

Furthermore, based on the screening of molecular dynamics simulation results, 2_703_6 exhibited more stable RMSD, indicating stronger intermolecular interactions between the minibinder and HER2, which contributed to the formation of a stable structure. This apparent affinity appears superior to that of 0_710_9, enabling the minibinder to bind more tightly to the HER2 target. In the two replicate simulations of 2_703_6, the RMSF values were nearly identical, which indicates that the structural flexibility was comparable and the simulations were representative. Meanwhile, in addition to directly blocking the functional sites of target proteins as demonstrated in this study, minibinders can also act as activators to induce signal activation [39]. Furthermore, studies have shown that minibinders can inhibit the entry of bacteria and viruses into cells through binding to them. These strategies provide a reference for the design of novel minibinders. In CAR-T studies, scFv-based CARs targeting the β subunit of voltage-gated sodium channels (VGSC) have shown feasibility in glioma preclinical models [40]. Because scFvs can self-associate and promote antigen-independent “tonic” signaling, small, monomeric binders (minibinders) are an attractive alternative recognition module [9]. Computationally designed minibinders typically exhibit high thermal stability and good solubility [41], and their reduced net positive charge and minimized exposed hydrophobic patches help limit nonspecific adsorption and self-aggregation [42], thereby potentially mitigating tonic signaling seen with some scFv-CARs. Importantly, the net level of tonic signaling still depends on the entire CAR architecture (hinge, transmembrane, and costimulatory domains), spacer design, and expression level.

However, the minibinders designed to date still face these challenges. Nevertheless, from a developability perspective, they offer several advantages over traditional antibodies. The “developability” of therapeutic molecules—essentially, their capacity to be developed into viable drugs—depends on key factors such as solubility, stability, manufacturability, and immunogenicity. These criteria are often used by pharmaceutical companies when deciding whether a candidate is truly druggable. While traditional antibodies exhibit high specificity and binding affinity, their relatively low stability and poor manufacturability pose significant challenges in their development [33]. In contrast, minibinders, as demonstrated in this study, show high solubility and structural stability—properties essential for scalable drug production. Furthermore, their small size allows for more straightforward synthesis, making them more manufacturable compared to full antibodies. Meanwhile, the minibinder we designed exhibits high stability and solubility, properties that are generally associated with low immunogenicity [43]. Additionally, our minibinder demonstrates strong binding affinity to the target, a characteristic that is also linked to reduced immunogenicity [44]. Therefore, we predict that 2_703_6 possesses low immunogenicity.

In summary, we have demonstrated that HER2-targeting minibinders, designed using the RFdiffusion method, show great promise as a next-generation therapeutic strategy. Their low molecular weight, high binding affinity, rapid production, and scalability position them as a potentially more efficient alternative to traditional antibodies. Moreover, the significant reduction in the time and resources required for their development underscores their translational potential. By addressing issues of solubility, stability, and manufacturability, minibinders could overcome many of the barriers faced by conventional antibody-based therapies, making them an attractive candidate for future therapeutic development. This work lays the foundation for the broader application of minibinders in HER2-targeted therapy and other clinical settings, with the potential to expand their utility beyond breast cancer to other tumor types.

This study has several limitations. First, cross-reactivity with domain IV of EGFR, HER3, and HER4 was not examined, so we cannot exclude contributions from these receptors in cellular assays. Second, surface-display levels in the whole-cell binding assay were not quantified, and negative signals may partly reflect expression or folding differences. Third, binding was assessed in two breast cancer cell lines without downstream signaling or growth-inhibition readouts; functional effects therefore remain to be established. Fourth, the binding epitope and complex geometry were not mapped; structural studies or mutational scanning will be required. Finally, in vivo delivery, pharmacokinetics, and immunogenicity were not evaluated.

## 5. Conclusions

In this study, we have successfully applied the RFdiffusion framework to de novo design minibinders targeting the trastuzumab epitope within domain IV of HER2. By integrating computational design, AlphaFold2-based structural validation, molecular dynamics simulations, and experimental assays, we have identified a minibinder (2_703_6) that exhibits nanomolar binding affinity, strong thermal stability, and target selectivity in our cellular assays. Compared with conventional monoclonal antibodies, the designed minibinder demonstrates a smaller molecular size, improved structural robustness, and ease of prokaryotic expression, thereby alleviating several limitations associated with antibody-based therapeutics. These findings highlight the potential of RFdiffusion-assisted design for the rapid generation of compact minibinders against clinically relevant membrane proteins. Importantly, the developed HER2 minibinder provides a promising foundation for future applications in breast cancer therapy, either as a direct therapeutic molecule or as a modular recognition domain for next-generation CAR T-cell engineering.

## Figures and Tables

**Table 1:** Amino acid sequences of the designed minibinders *.

Number	Sequence	Molecular Weight (kDa)
0_293_7	AREAARQAAIDEISALFARARANPDLSPAERAALAAAITAAYRRYRALL	6.12
0_324_7	SAAEAATRARIRARAAEVAEQIKDLSPEERKALYLQAFQEFSGESQRQLNLLHSLLNERWAELQ	8.45
0_459_7	STAAAIAAARAEVENLPYEEALARADELAERLGYEKRTVNGGTIYLPA	5.95
0_679_6	AERQQKIAQLKALLATAEGYLKLLKDNEEAQIRVAEHLDKQLAEVLGEKEVKEGITLEEMIARIQAEIAKLEA	9.48
0_710_3	AAQTAALIAQIATLNAAQNIALISQLFAAARSDPSLDRAELATQITAAYQQYRALL	6.88
0_710_5	SAETEATIERIKTMNREENIELISEAFARARSDPSLDRAALARIITAAYQQYRKLL	7.39
0_710_6	SAATAATIARIATLNAEENIALISQLFAQARTDPSLDRAALATQITAAYRQYRALL	6.95
0_710_7	SEETAATIARIETMTAEENIALISELFERARTDPSLDRAELATMITAAVQRYRRLL	7.30
0_710_9	DAATAATIARIATLDAAGNIALISELFAQARSDPSLDRAALARQITAAYQRYRALL	6.89
0_818_7	SEALLARLREVAATKSDEEFARELARVDAEGLGREAAREAFRERAASLPTNAAVNRLMALYSEALAEERARLAAEAAAA	9.89
0_1007_6	AAKAAQIATLNAIRDALKAGDLETAKALIAATSLSNTVANHVLNALSYAERAKADPATAAADAAEMDAEFAKALAAVNAE	9.42
0_1156_1	MKVVVLPASAPVEEKRRVAQELVAQYGSLVVTTVDDVPVEEAVAIAKRDIAAIKRAYPQVKAILVITSDEAAKALKA	9.58
1_250_0	RAALLERAITAFDLAAYAVRQGNRAAAAQALAHALALLEEAGLAVSEELRRLLELPPEEALALLARERARLRAL	9.29
1_394_0	PVHVPLSAVASTPEELRARVREIVAEFSIEEVTASALRIQPLLPPELFAAILATLEELREEAEKA	8.27
1_968_7	ARYRVTFHDTGLTYETDKAAAERDVALAKEKGLRVTVEPVVT	5.42
1_1124_9	PHHRQLPAAELRALAQAELARAQAAGQVAQAQRHQAILAQVDAGQPLTYIALPP	6.68
2_128_0	PLHVLIFKPGKVLYRVETPEITREAEIDLESALALTRVLVRAGARVVVENGEEALKEAEKTNKKDAELIREILELVK	10.03
2_128_4	PLIVVELRPGRVLLTVVTPTITKTAEIDLESALDLVRVLKRAGVKVEVRGGDAALALAKRTDPAAAALIEAILAEVA	9.39
2_128_8	ALHVVRVEPGRITLVTRTPTITRSAEIDAESALALVRVLRRAGATVELENLDAGIAAAAATDPATAALLRAIAAEAA	9.22
2_337_7	ARVEATVSFPDREAAYAVLREVNVRFIVDFQPDGTVVLIAPESDRAGLEAEAAKLKALVAARA	7.87
2_520_2	RWVYRELTVAELEALRAREASPVDQPRWDAVLAQMRASGQPALYVQRLP	6.53
2_520_5	RWVYRELTVEELKAIAARETLPVDAPRWAAVLAQAEASGQPVWYVRRLP	6.50
2_645_0	TVLSPEELIRRLYRRAAELIATLPPSVQLTVAVLATPTSVEVRVTAPAEAAAHAEALRAELEAEIA	8.19
2_703_2	VVNVELSPELLALQEQIQALADRISELFARARAATDPAEKARLRAEITAAILQRRALIAQRRALLAA	8.53
2_703_6	VVNVELSPELLALEAEIQALADQISAAFAAARAETDPAAKAALRAQITALIQQRRALIAQRRALLAA	8.24
3_15_0	SATRELNRLLGEASLFIEELRKRAPELSPEELKKILEEKVEEYAKINPLVAAYMKHQVEKLL	8.31
3_870_2	MTPEELEALRARILAMSPEEIRALFRADAQLAAAALEAANLAAAAAAGLTVQGGVVRPEP	7.22
3_1069_8	MSRFTEKLLALYRTNVAAYTAAGNAANIEKVTAYYKAEAASLPDAAEVNAELDAAKAAALAALAAA	7.92
4_114_3	PVLRHRTITEAEALAITARTAATLSPLQAAALRARTAALLAAARAAGTPLTYISLE	6.76
4_114_4	PRLVHREVTEAEAQAILAATAASLSPLQAALLAQRHAAILAQARAAGLPLTYTSLE	6.81
4_1157_3	MRRVVLDRLAPGRWRLTVTTPERTLVFELSTEVARALWRRYTADEIAARPEALVAEALAAA	7.99

* The table lists the primary sequences of the 31 minibinders generated through the de novo design pipeline. All minibinders are predominantly α-helical and were selected based on structural confidence and binding stability scores (pLDDT > 70, pAE_interaction < 10).

## Data Availability

The data that support the findings of this study are available in the methods and/or Supplementary Material of this article. Materials are available on request from the corresponding author.

## Supplementary Materials

The following supporting information can be downloaded at: https://www.mdpi.com/article/10.3390/biom15111587/s1, Figure S1: Analysis of hydrophobic residues in HER2 domain IV; Figure S2: Predicted structural models of designed minibinders in complex with the target protein generated by AlphaFold2; Figure S3: PAE_int and pLDDT scores of the minibinder predicted by AlphaFold2; Figure S4: Flow cytometry analysis of target binding by surface-displayed minibinders on *E. coli* E; Figure S5: Plasmid map of 2_703_6 construct; Figure S6: MD simulations of HER2 IV–0_710_9 complexes; Figure S7: HT spectra corresponding to the CD measurements; Table S1: Strains and plasmids used in this study; Table S2: Primers used in this study.

## References

[1] Cheng X. (2024). A Comprehensive Review of HER2 in Cancer Biology and Therapeutics. Genes.

[2] Chao W.R., Lee M.Y., Lee Y.J., Sheu G.T., Chiu H.H., Shen H.P., Han C.P. (2025). Profiling of HER2, KRAS, and PIK3CA mutations in uterine cervical neuroendocrine carcinoma and implications for oncogenic driver targeting therapy. Cancer Genet..

[3] Di Lisa D., Cortese K., Chiappalone M., Arnaldi P., Martinoia S., Castagnola P., Pastorino L. (2024). Electrophysiological and morphological modulation of neuronal-glial network by breast cancer and nontumorigenic mammary cell conditioned medium. Front. Bioeng. Biotechnol..

[4] Wilcock D.M., Zhao J., Gulbahce H.E. (2025). Resolving HER2 status in breast carcinoma patients with complete deletion of CEP17 in fluorescence in-situ hybridization assays. Cancer Genet..

[5] Tapia M., Hernando C., Martínez M.T., Burgués O., Tebar-Sánchez C., Lameirinhas A., Ágreda-Roca A., Torres-Ruiz S., Garrido-Cano I., Lluch A. (2023). Clinical Impact of New Treatment Strategies for HER2-Positive Metastatic Breast Cancer Patients with Resistance to Classical Anti-HER Therapies. Cancers.

[6] Martínez Bedoya D., Dutoit V., Migliorini D. (2021). Allogeneic CAR T Cells: An Alternative to Overcome Challenges of CAR T Cell Therapy in Glioblastoma. Front. Immunol..

[7] Han X., Cinay G.E., Zhao Y., Guo Y., Zhang X., Wang P. (2017). Adnectin-Based Design of Chimeric Antigen Receptor for T Cell Engineering. Mol. Ther..

[8] Krokhotin A., Du H., Hirabayashi K., Popov K., Kurokawa T., Wan X., Ferrone S., Dotti G., Dokholyan N.V. (2019). Computationally Guided Design of Single-Chain Variable Fragment Improves Specificity of Chimeric Antigen Receptors. Mol. Ther. Oncolytics.

[9] Landoni E., Fucá G., Wang J., Chirasani V.R., Yao Z., Dukhovlinova E., Ferrone S., Savoldo B., Hong L.K., Shou P. (2021). Modifications to the Framework Regions Eliminate Chimeric Antigen Receptor Tonic Signaling. Cancer Immunol. Res..

[10] Zahid R., Wang J., Cai Z., Ishtiaq A., Liu M., Ma D., Liang Y., Xu Y. (2024). Single chain fragment variable, a new theranostic approach for cardiovascular diseases. Front. Immunol..

[11] Kim T.Y., Cha J.S., Kim H., Choi Y., Cho H.-S., Kim H.-S. (2021). Computationally-guided design and affinity improvement of a protein binder targeting a specific site on HER2. Comput. Struct. Biotechnol. J..

[12] Xia Z., Jin Q., Long Z., He Y., Liu F., Sun C., Liao J., Wang C., Wang C., Zheng J. (2024). Targeting overexpressed antigens in glioblastoma via CAR T cells with computationally designed high-affinity protein binders. Nat. Biomed. Eng..

[13] Wei L., Hu Y., Liu Y., Xing B., Wang K., Weng J., Liu Z., Fang Y., Ming K., Mei M. (2025). De novo design mini-binder proteins targeting the glycoproteins D to inhibit PRV replication in PK15 cells. Int. J. Biol. Macromol..

[14] Berger S., Seeger F., Yu T.Y., Aydin M., Yang H., Rosenblum D., Guenin-Macé L., Glassman C., Arguinchona L., Sniezek C. (2024). Preclinical proof of principle for orally delivered Th17 antagonist miniproteins. Cell.

[15] Wang Y., Liu L., Yang Q.Y., Yu K. (2025). Novel anti-HER2 nanobody-drug conjugates with enhanced penetration of solid tumor and BBB, reduced systemic exposure and superior antitumor efficacy. Acta Pharmacol. Sin..

[16] Watson J.L., Juergens D., Bennett N.R., Trippe B.L., Yim J., Eisenach H.E., Ahern W., Borst A.J., Ragotte R.J., Milles L.F. (2023). De novo design of protein structure and function with RFdiffusion. Nature.

[17] Pacesa M., Nickel L., Schellhaas C., Schmidt J., Pyatova E., Kissling L., Barendse P., Choudhury J., Kapoor S., Alcaraz-Serna A. (2025). BindCraft: One-shot design of functional protein binders. bioRxiv.

[18] Vázquez Torres S., Leung P.J.Y., Venkatesh P., Lutz I.D., Hink F., Huynh H.H., Becker J., Yeh A.H., Juergens D., Bennett N.R. (2024). De novo design of high-affinity binders of bioactive helical peptides. Nature.

[19] Liu Z., Yang Y., Chen M., Chen X., Ming K., Liu Y., Weng J., Xing B., Wei L., Wang Z. (2025). De novo designed mini-binders targeting glyceraldehyde-3-phosphate dehydrogenase of Streptococcus equi ssp. zooepidemicus provided partial protection in mice model of infection. Int. J. Biol. Macromol..

[20] Glögl M., Krishnakumar A., Ragotte R.J., Goreshnik I., Coventry B., Bera A.K., Kang A., Joyce E., Ahn G., Huang B. (2024). Target-conditioned diffusion generates potent TNFR superfamily antagonists and agonists. Science.

[21] Bennett N.R., Coventry B., Goreshnik I., Huang B., Allen A., Vafeados D., Peng Y.P., Dauparas J., Baek M., Stewart L. (2023). Improving de novo protein binder design with deep learning. Nat. Commun..

[22] Wilkins M.R., Gasteiger E., Bairoch A., Sanchez J.C., Williams K.L., Appel R.D., Hochstrasser D.F. (1999). Protein identification and analysis tools in the ExPASy server. Methods Mol. Biol..

[23] Kyte J., Doolittle R.F. (1982). A simple method for displaying the hydropathic character of a protein. J. Mol. Biol..

[24] Arai S., Suzuki H. (2023). Immobilization of *E. coli* expressing gamma-glutamyltranspeptidase on its surface for gamma-glutamyl compound production. AMB Express.

[25] Lei S., Zheng R., Zhang S., Chen R., Wang S., Sun K., Zeng H., Wei W., He J. (2021). Breast cancer incidence and mortality in women in China: Temporal trends and projections to 2030. Cancer Biol. Med..

[26] Shagufta, Ahmad I., Nelson D.J., Hussain M.I., Nasar N.A. (2024). Potential of covalently linked tamoxifen hybrids for cancer treatment: Recent update. RSC Med. Chem..

[27] Jordan V.C. (1995). Tamoxifen: Toxicities and drug resistance during the treatment and prevention of breast cancer. Annu. Rev. Pharmacol. Toxicol..

[28] Bisht A., Avinash D., Sahu K.K., Patel P., Das Gupta G., Kurmi B.D. (2025). A comprehensive review on doxorubicin: Mechanisms, toxicity, clinical trials, combination therapies and nanoformulations in breast cancer. Drug Deliv. Transl. Res..

[29] Gautam S., Maurya R., Vikal A., Patel P., Thakur S., Singh A., Gupta G.D., Kurmi B.D. (2025). Understanding drug resistance in breast cancer: Mechanisms and emerging therapeutic strategies. Med. Drug Discov..

[30] Jallah J.K., Dweh T.J., Anjankar A., Palma O. (2023). A Review of the Advancements in Targeted Therapies for Breast Cancer. Cureus.

[31] Swain S.M., Shastry M., Hamilton E. (2023). Targeting HER2-positive breast cancer: Advances and future directions. Nat. Rev. Drug Discov..

[32] Kunte S., Abraham J., Montero A.J. (2020). Novel HER2-targeted therapies for HER2-positive metastatic breast cancer. Cancer.

[33] Cao L., Goreshnik I., Coventry B., Case J.B., Miller L., Kozodoy L., Chen R.E., Carter L., Walls A.C., Park Y.-J. (2020). De novo design of picomolar SARS-CoV-2 miniprotein inhibitors. Science.

[34] Sonkin D., Thomas A., Teicher B.A. (2024). Cancer treatments: Past, present, and future. Cancer Genet..

[35] Cao L., Coventry B., Goreshnik I., Huang B., Sheffler W., Park J.S., Jude K.M., Marković I., Kadam R.U., Verschueren K.H.G. (2022). Design of protein-binding proteins from the target structure alone. Nature.

[36] Zheng M., Li C., Zhou M., Jia R., She F., Wei L., Cheng F., Li Q., Cai J., Wang Y. (2021). Peptidomimetic-based antibody surrogate for HER2. Acta Pharm. Sin. B.

[37] Rodak M., Dekempeneer Y., Wojewodzka M., Caveliers V., Covens P., Miller B.W., Sevenois M.B., Bruchertseifer F., Morgenstern A., Lahoutte T. (2022). Preclinical Evaluation of 225Ac-Labeled Single-Domain Antibody for the Treatment of HER2pos Cancer. Mol. Cancer Ther..

[38] Oroujeni M., Westerlund K., Papalanis E., van Deventer A., Liu Y., Clinton J., Wang Z., Zelepukin I., Orlova A., Tolmachev V. (2025). Designed Ankyrin Repeat Protein-Mediated Peptide Nucleic Acid-Based Pretargeting: A Proof-of-Principle Study. J. Nucl. Med..

[39] Adams C.S., Kim H., Burtner A.E., Lee D.S., Dobbins C., Criswell C., Coventry B., Tran-Pearson A., Kim H.M., King N.P. (2025). De novo design of protein minibinder agonists of TLR3. Nat. Commun..

[40] Liu H., Hamaia S.W., Dobson L., Weng J., Hernández F.L., Beaudoin C.A., Salvage S.C., Huang C.L., Machesky L.M., Jackson A.P. (2025). The voltage-gated sodium channel β3 subunit modulates C6 glioma cell motility independently of channel activity. Biochim. Biophys. Acta Mol. Basis Dis..

[41] Weng J., Geng M., Hu X., Hu Y., Yang Y., Xing B., Wu Z., Wei Z. (2025). Design of minibinder proteins specific to TNFR1. Int. J. Biol. Macromol..

[42] Chen J., Qiu S., Li W., Wang K., Zhang Y., Yang H., Liu B., Li G., Li L., Chen M. (2023). Tuning charge density of chimeric antigen receptor optimizes tonic signaling and CAR-T cell fitness. Cell Res..

[43] Snapper C.M. (2018). Distinct Immunologic Properties of Soluble Versus Particulate Antigens. Front. Immunol..

[44] Jensen K.K., Andreatta M., Marcatili P., Buus S., Greenbaum J.A., Yan Z., Sette A., Peters B., Nielsen M. (2018). Improved methods for predicting peptide binding affinity to MHC class II molecules. Immunology.

