# Supplementary material for: De Novo Design of High-Affinity HER2-Targeting Protein Minibinders
Source: Biomolecules. 2025 Nov 12;15(11):1587. doi: 10.3390/biom15111587 (PMC12650588; doi:10.3390/biom15111587)
Supplement: Supplementary file 1 [file biomolecules-15-01587-s001.zip › Supplementary Materials.pdf]

De novo designed high-affinity protein minibinder on HER2

Yize Zhao[1], Wenping Wei[1], Zijun Cheng[1], Min Yang[1], Yunjun Yan[1]*

[1]Key Laboratory of Molecular Biophysics of the Ministry of Education, College of Life Science and Technology, Huazhong University of Science and Technology, Wuhan, China.

*To whom correspondence should be addressed:
Yunjun Yan, E-mail: yanyunjun@hust.edu.cn

Supplementary Materials

Table S1 Strains and plasmids used in this study

Table S2 Primers used in this study

Figure S1. Analysis of hydrophobic residues in HER2 domain IV

Figure S2. Predicted structural models of designed minibinders in complex with the target protein generated by AlphaFold2.

Figure S3. PAE_int and pLDDT scores of the minibinder predicted by AlphaFold2.

Figure S4. Flow cytometry analysis of target binding by surface-displayed minibinders on *E. coli* E.

Figure S5. Plasmid map of 2_703_6 construct

Figure S6. MD simulations of HER2 IV–0_710_9 complexes

Figure S7. HT spectra corresponding to the CD measurements

Table S1 Strains and plasmids used in this study

**Strains and plasmids**	Relevant genotype or features	Source or reference
Strain		
DH5α	Host for plasmid construction	Novagen
BL21	Host for overexpression	Novagen
YiaT	Ec outer membrane anchoring protein used for surface display	Novagen
GFP_HER2	Ec/pET28a-GFP_HER2_IV BL21(DE3) with pET28a-GFP_HER2_IV, GFP-HER2_IV fusion protein-expressing strain	This study
YiaT_0_239_7	Ec/YiaT_0_239_7 BL21(DE3) with YiaT_0_239_7, YiaT_0_239_7 surface display strain	This study
YiaT_0_324_7	Ec/YiaT_0_324_7 BL21(DE3) with YiaT_0_324_7, YiaT_0_324_7 surface display strain	This study
YiaT_0_459_7	Ec/YiaT_0_459_7 BL21(DE3) with YiaT_0_459_7, YiaT_0_459_7 surface display strain	This study
YiaT_0_679_6	Ec/YiaT_0_679_6 BL21(DE3) with YiaT_0_679_6, YiaT_0_679_6 surface display strain	This study
YiaT_0_710_3	Ec/YiaT_0_710_3 BL21(DE3) with YiaT_0_710_3, YiaT_0_710_3 surface display strain	This study
YiaT_0_710_5	Ec/YiaT_0_710_5 BL21(DE3) with YiaT_0_710_5, YiaT_0_710_5 surface display strain	This study
YiaT_0_710_6	Ec/YiaT_0_710_6 BL21(DE3) with YiaT_0_710_6, YiaT_0_710_6 surface display strain	This study
YiaT_0_710_7	Ec/YiaT_0_710_7 BL21(DE3) with YiaT_0_710_7, YiaT_0_710_7 surface display strain	This study
YiaT_0_710_9	Ec/YiaT_0_710_9 BL21(DE3) with YiaT_0_710_9, YiaT_0_710_9 surface display strain	This study
YiaT_0_818_7	Ec/YiaT_0_818_7 BL21(DE3) with YiaT_0_818_7, YiaT_0_818_7 surface display strain	This study
YiaT_0_1007_6	Ec/YiaT_0_1007_6 BL21(DE3) with YiaT_0_1007_6, YiaT_0_1007_6 surface display strain	This study
YiaT_1_250_0	Ec/YiaT_1_250_0 BL21(DE3) with YiaT_1_250_0, YiaT_1_250_0 surface display strain	This study
YiaT_1_394_0	Ec/YiaT_1_394_0 BL21(DE3) with YiaT_1_394_0, YiaT_1_394_0 surface display strain	This study
YiaT_1_968_7	Ec/YiaT_1_968_7 BL21(DE3) with YiaT_1_968_7, YiaT_1_968_7 surface display strain	This study
YiaT_2_128_0	Ec/YiaT_2_128_0 BL21(DE3) with YiaT_2_128_0, YiaT_2_128_0 surface display strain	This study
YiaT_2_128_8	Ec/YiaT_2_128_8 BL21(DE3) with YiaT_2_128_8, YiaT_2_128_8 surface display strain	This study
YiaT_2_520_2	Ec/YiaT_2_520_2 BL21(DE3) with YiaT_2_520_2, YiaT_2_520_2 surface display strain	This study

YiaT_2_520_5	Ec/YiaT_2_520_5	BL21(DE3) with YiaT_2_520_5, YiaT_2_520_5 surface display strain	This study
YiaT_2_645_0	Ec/YiaT_2_645_0	BL21(DE3) with YiaT_2_645_0, YiaT_2_645_0 surface display strain	This study
YiaT_2_703_2	Ec/YiaT_2_703_2	BL21(DE3) with YiaT_2_703_2, YiaT_2_703_2 surface display strain	This study
YiaT_2_703_6	Ec/YiaT_2_703_6	BL21(DE3) with YiaT_2_703_6, YiaT_2_703_6 surface display strain	This study
YiaT_4_114_3	Ec/YiaT_4_114_3	BL21(DE3) with YiaT_4_114_3, YiaT_4_114_3 surface display strain	This study
YiaT_4_114_4	Ec/YiaT_4_114_4	BL21(DE3) with YiaT_4_114_4 YiaT_4_114_4 surface display strain	This study
YiaT_4_1157_3	Ec/YiaT_4_1157_3	BL21(DE3) with YiaT_4_1157_3, YiaT_4_1157_3 surface display strain	This study
GFP_2_703_6	Ec/pET28a-GFP_2_703_6	BL21(DE3) with pET28a-GFP_2_703_6, GFP-2_703_6 fusion protein-expressing strain	This study
2_703_6	Ec/pET28a-2_703_6	BL21(DE3) with pET28a- 2_703_6, 2_703_6 fusion protein-expressing strain	This study

Table S2 Primers used in this study

Name	Sequence (5’-3’)
F_GFP	GGCCTGGTGCCGCGCGGCAGCCATATGATGCGTAAAGGCGAGGAACTG
R_HER2_GFP	TACACATTCATCTTCAGGACGTTTATACAGTTCATCCATACC
HER2_F	GGTATGGATGAACTGTATAAACGTCCTGAAGATGAATGTGTA
HER2_R	AGTC**TCTAGA**TTAGCCCAGCGGTGCCATAT
Com_F	GGCCTGGTGCCGCGCGGCAGC
Com_R	TTGTCGACGGAGCTCGAATTC
YiaT_F	CCTGGTGCCGCGCGGCAGCCA
YiaT_R	ATGTTCCTATTAATTAACATG
YiaT-BIND-F	GTGCCGCGCGGCAGCCATATGATGTTAATTAATAGGAACAT
YiaT_6LBX_R	GGGTGCTCACGGTGATTGTTTCACGATCAATCATAGGGCTGT
YiaT_0_239_7_R	CTGTCTTGCTGCTTCACGTGCACGATCAATCATAGGGCTGT
YiaT_0_324_7_R	GGGTTGCTGCTTCTGCTGCGCTACGATCAATCATAGGGCTGT
YiaT_0_459_7_R	TGCAATTGCTGCTGCGGTGCTACGATCAATCATAGGGCTGT
YiaT_0_679_6_R	CAATTTTCTGCTGACGTTCTGCACGATCAATCATAGGGCTGT
YiaT_0_710_3_R	TAATGCTGCGGTCTGTGCTGCACGATCAATCATAGGGCTGT
YiaT_0_710_5_R	TGTTGCTTCTGTTTCTGCGCTACGATCAATCATAGGGCTGT
YiaT_0_710_6_R	TGGTTGCTGCTGTTGCTGCGCTACGATCAATCATAGGGCTGT
YiaT_0_710_7_R	TTGTTGCTGCTGTTTCTTCGCTACGATCAATCATAGGGCTGT
YiaT_0_710_9_R	TGGTCGCCGCGGTTGCTGCATCACGATCAATCATAGGGCTGT
YiaT_0_818_7_R	GTCTTGCTAACAGTGCTTCGCTACGATCAATCATAGGGCTGT
YiaT_0_1007_6_R	CAATCTGTGCTGCTTTTGCTGCACGATCAATCATAGGGCTGT
YiaT_1_250_0_R	CTCTTTCTAATAATGCTGCACGACGATCAATCATAGGGCTGT
YiaT_1_394_0_R	CAGAAAGAGGAACATGAACAGGACGATCAATCATAGGGCTGT
YiaT_1_968_7_R	GAAAGGTAACTCTATAACGTGCACGATCAATCATAGGGCTGT
YiaT_2_128_0_R	TGAAGATAAGAACATGTAAAGGACGATCAATCATAGGGCTGT
YiaT_2_128_8_R	CAACTCTAACAACATGCAGTGCACGATCAATCATAGGGCTGT
YiaT_2_520_2_R	TTAATTCACGATAAACCCAACGACGATCAATCATAGGGCTGT
YiaT_2_520_5_R	TTAATTCACGATAAACCCAACGACGATCAATCATAGGGCTGT
YiaT_2_645_0_R	GTTCTTCTGGGCTCAGCACGGTACGATCAATCATAGGGCTGT
YiaT_2_703_2_R	GACTCAGCTCAACGTTAACAACACGATCAATCATAGGGCTGT
YiaT_2_703_6_R	GACTCAGCTCAACGTTAACAACACGATCAATCATAGGGCTGT
YiaT_4_114_3_R	GGTACGATGACGAAGAACAGGACGATCAATCATAGGGCTGTC
YiaT_4_114_4_R	TTCACGATGAACAAGACGAGGACGATCAATCATAGGGCTGTC
YiaT_4_1157_3_R	GTCCAGAACAACACGGCGCATACGATCAATCATAGGGCTGTC

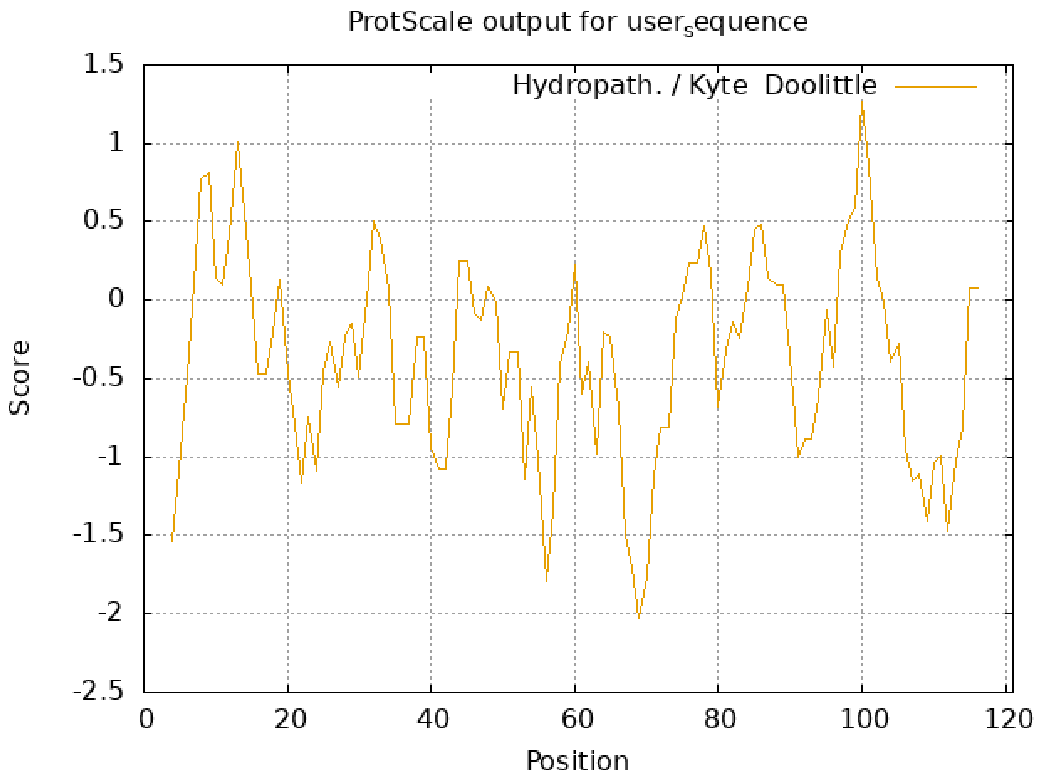

Supplementary Figure S1. Analysis of hydrophobic residues in HER2 domain IV. The hydropathy profile of HER2 domain IV was calculated using the ProtScale tool (ExPASy) with the Kyte–Doolittle method. Positive values indicate hydrophobic regions, while negative values represent hydrophilic regions. The analysis was performed to identify the distribution of hydrophobic residues along the amino acid sequence, which may contribute to domain-specific structural and functional characteristics.

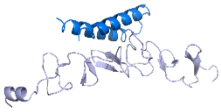
0_239_7

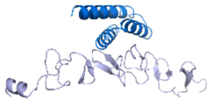
0_324_7

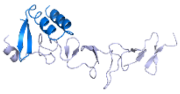
0_459_7

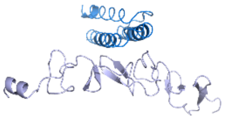
0_679_6

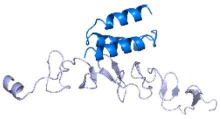
0_710_3

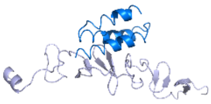
0_710_5

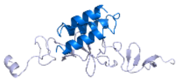
0_710_6

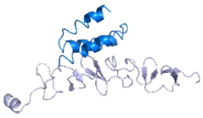
0_710_7

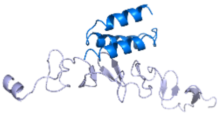
0_710_9

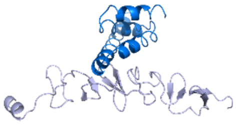
0_818_7

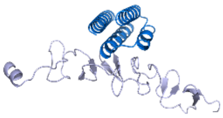
0_1007_6

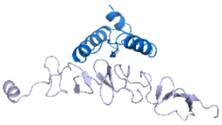
0_1156_1

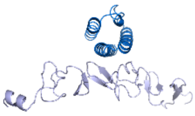
1_250_0

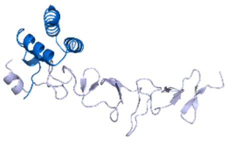
1_394_0

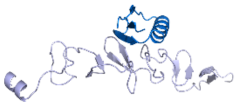
1_968_7

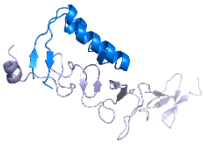
1_1124_9

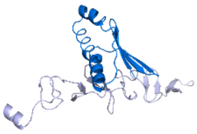
2_128_0

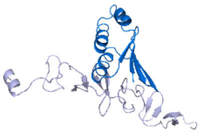
2_128_4

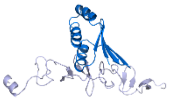
2_128_8

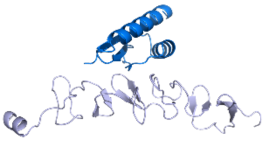
2_337_7

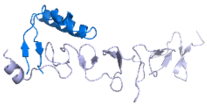
2_520_2

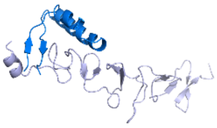
2_520_5

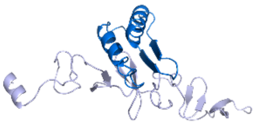
2_645_0

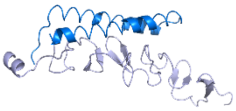
2_703_2

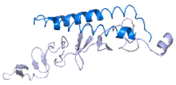
2_703_6

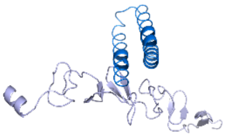
3_15_0

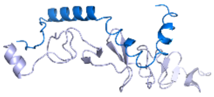
3_870_2

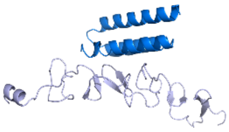
3_1069_8

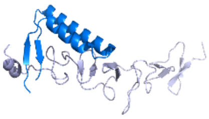
4_114_3

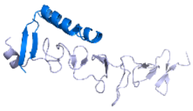
4_114_4

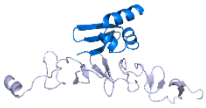
4_1157_3

Supplementary Figure S2. Predicted structural models of designed minibinders in complex with the target protein generated by AlphaFold2. Multiple designed minibinder candidates were modeled using AlphaFold2 in complex with the target protein. In each panel, the minibinder is shown in blue and the target protein in light purple. Design identifiers are indicated below each model.

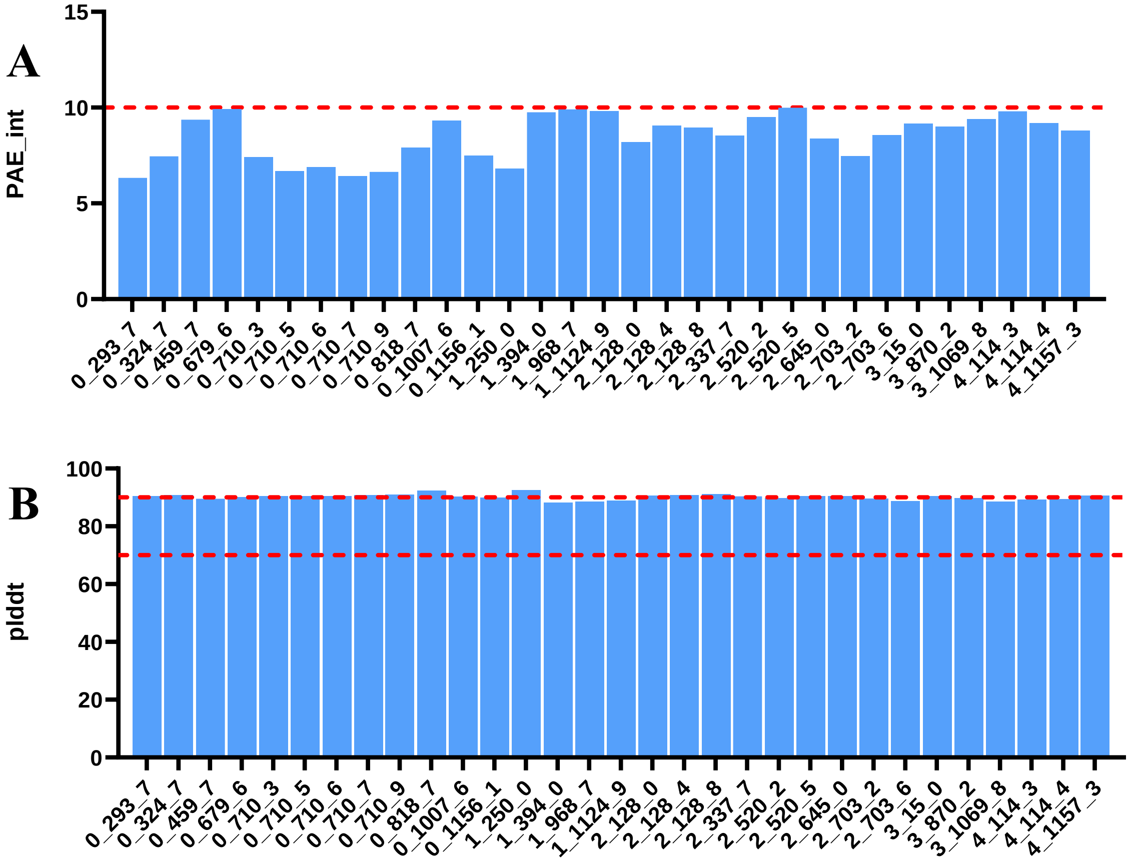

Supplementary Figure S3.PAE_int and pLDDT scores of the minibinder predicted by AlphaFold2. (A) PAE_int plot showing per-residue average PAE values. The red dashed line (10 Å) indicates the selection threshold; designs with values below this threshold were considered to have high-confidence domain orientation predictions. **(B)** pLDDT scores for each residue. The red dashed lines represent the thresholds for high confidence (⩾ 90, upper line) and acceptable confidence (⩾ 70, lower line). Designs with all residues above the lower threshold and most residues above the upper threshold were selected for further analysis. These criteria were used to screen and select high-quality structural models from the design pool.

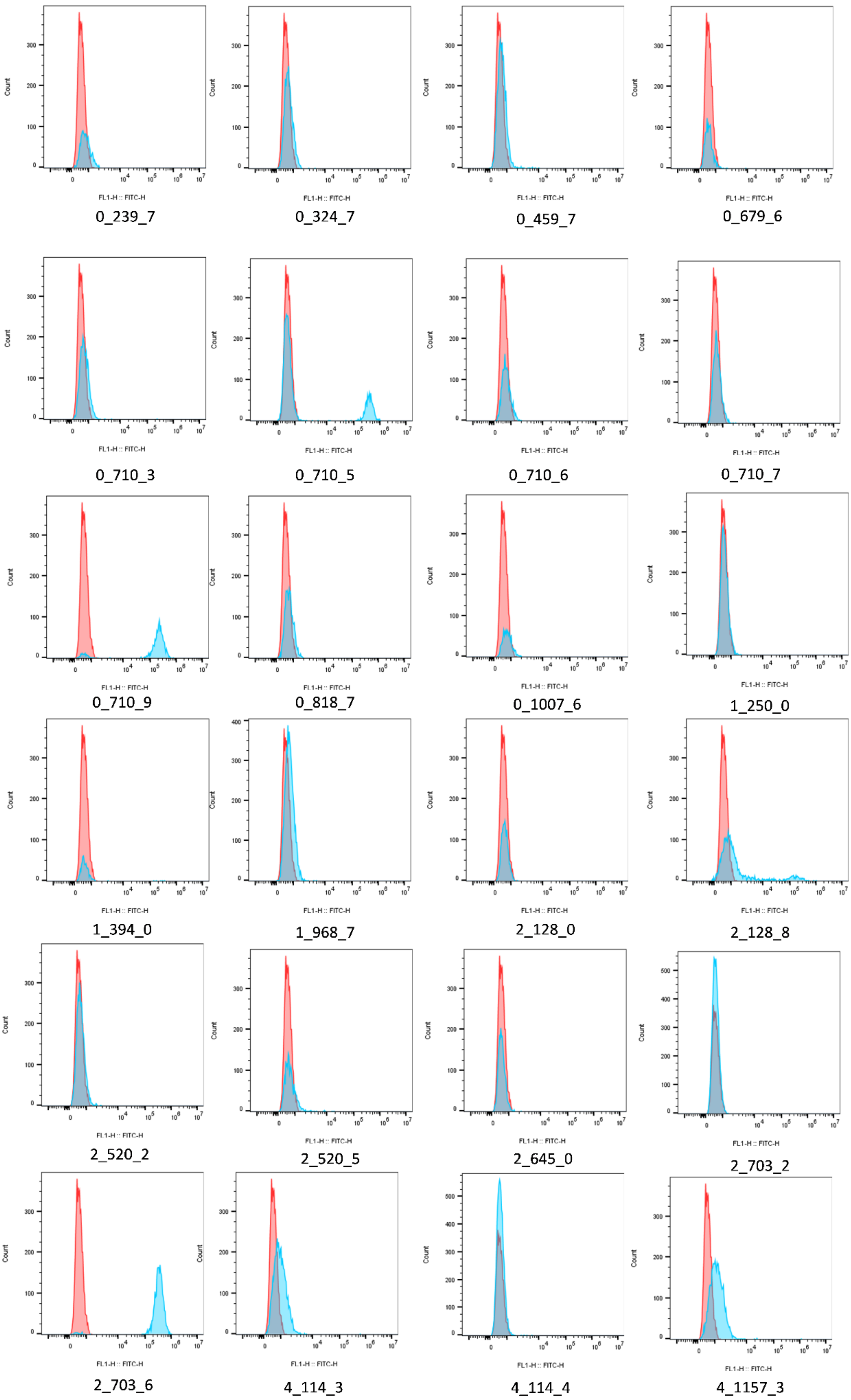
Count
FL1-H :: FITC-H
0_239_7
0_324_7
0_459_7
0_679_6
0_710_3
0_710_5
0_710_6
0_710_7
0_710_9
0_818_7
0_1007_6
1_250_0
1_394_0
1_968_7
2_128_0
2_128_8
2_520_2
2_520_5
2_645_0
2_703_2
2_703_6
4_114_3
4_114_4
4_1157_3

Supplementary Figure S4. **Flow cytometry analysis of target binding by surface-displayed minibinders on *E. coli.*** *E. coli* strains expressing surface-displayed minibinders were incubated overnight at 4 °C with GFP-labeled target protein. After incubation, cells were washed three times with PBS to remove unbound protein and analyzed by flow cytometry. In each histogram, the red peak represents the negative control (*E. coli* with empty display vector), and the blue peak represents the corresponding minibinder-displaying strain. A rightward shift in fluorescence intensity relative to the control indicates successful target binding. Design identifiers are shown below each histogram.

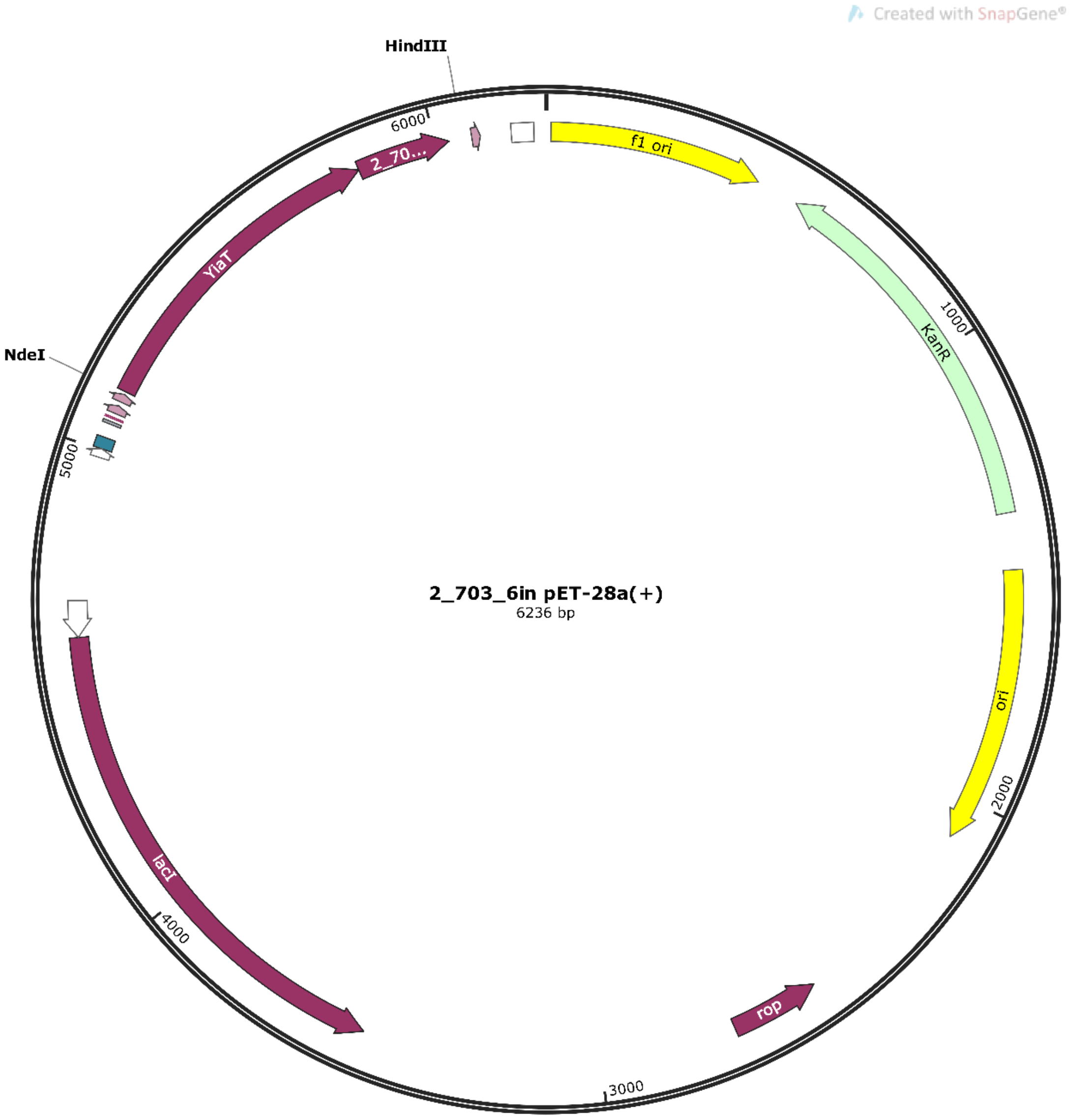

Figure S5. Plasmid map of 2_703_6 construct

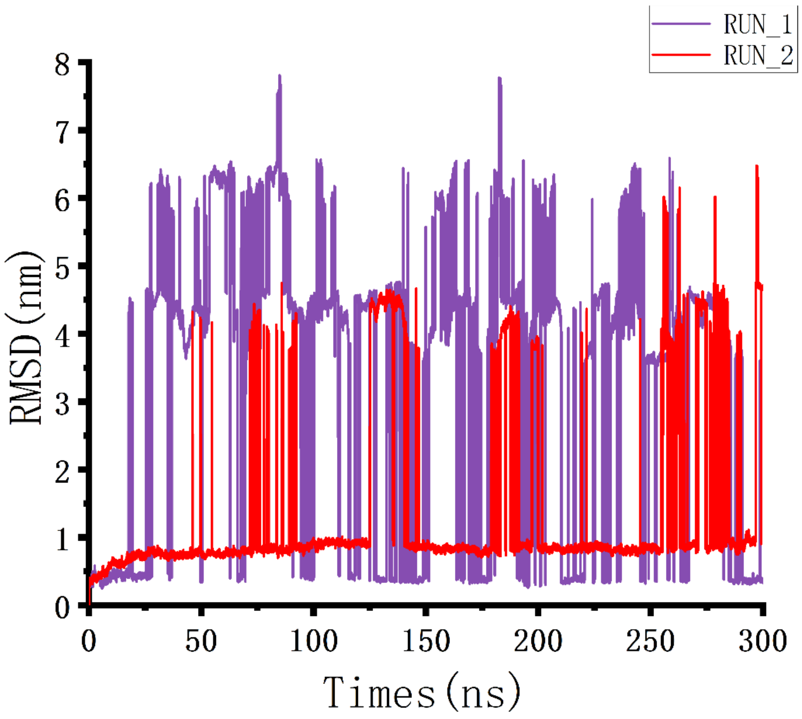

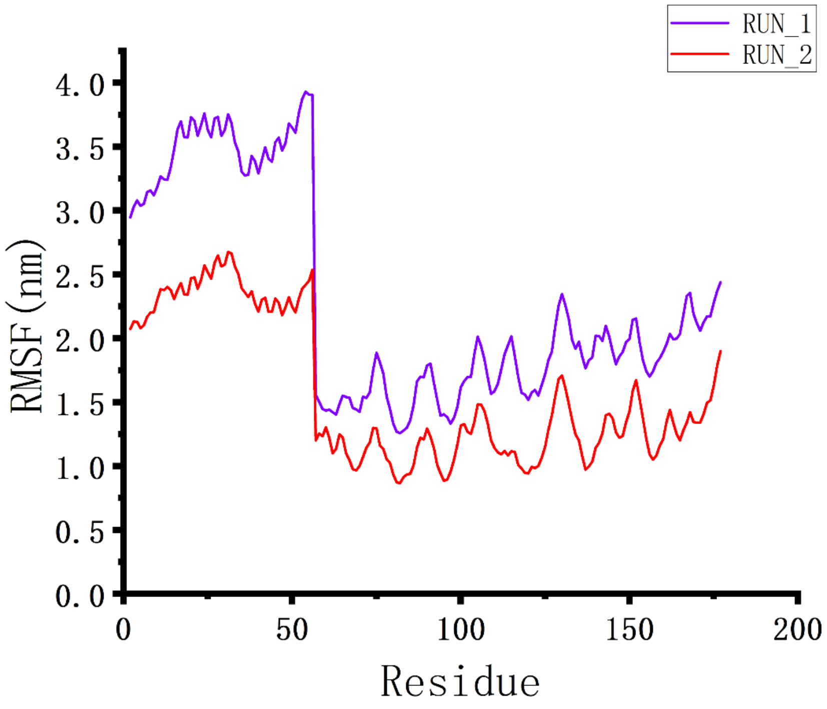

Supplementary Figure S6. MD simulations of HER2 IV–0_710_9 complexes RMSD and RMSF profiles of the HER2 IV–0_710_9 complex over 300 ns. Residues 1–67 correspond to the binder, and residues 68–188 represent HER2 domain IV.

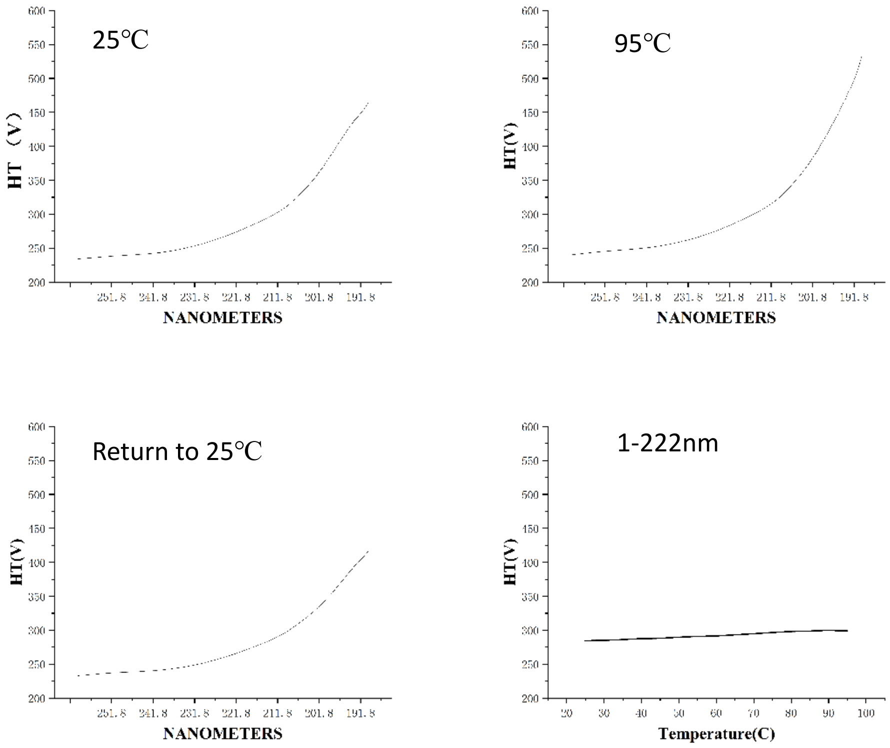

Figure S7. HT spectra corresponding to the CD measurements
