# Supplementary material for: De Novo Design of High-Affinity HER2-Targeting Protein Minibinders
Source: Biomolecules. 2025 Nov 12;15(11):1587. doi: 10.3390/biom15111587 (PMC12650588; doi:10.3390/biom15111587)
Supplement: Supplementary file 1 [file biomolecules-15-01587-s001.zip › Supplementary Materials2.pdf]

S1. Software, Versions, Sources

Tool/Package	Version	Git commit	Source/ URL
RFdiffusion	v1.1.0	fa34014	https://github.com/RosettaCommons/RFdiffusion
ProteinMPNN(dl_binder_design)	v1.0.0	56944f4	https://github.com/nrbennet/dl_binder_design
AlphaFold2(dl_binder_design)	v1.0.0	1644c74	https://github.com/nrbennet/dl_binder_design
Rosetta	v3.14	dd237bf	https://github.com/RosettaCommons/main

S2.Structural Inputs & Pre-processing

/home/zyz/miniconda3/bin/python /mnt/Software/rosetta314/main/tools/protein_tools/scripts/clean_pdb.py 1N8Z-D3.pdb C

S3. RFdiffusion: Backbone Generation

0

./scripts/run_inference.py inference.output_prefix=example_outputs/1N8Zdesign0/ inference.input_pdb=examples/input_pdbs/1N8Z-D3_C.pdb 'contigmap.contigs=[C1-121/0 40-80]' 'ppi.hotspot_res=[A11,A19,A34,A45,A75,A76,A79]' inference.num_designs=1200 denoiser.noise_scale_ca=0 denoiser.noise_scale_frame=0

0.1

./scripts/run_inference.py inference.output_prefix=example_outputs/1N8Zdesign0.1/ inference.input_pdb=examples/input_pdbs/1N8Z-D3_C.pdb 'contigmap.contigs=[C1-121/0 40-80]' 'ppi.hotspot_res=[A11,A19,A34,A45,A75,A76,A79]' inference.num_designs=1200 denoiser.noise_scale_ca=0.1 denoiser.noise_scale_frame=0.1

0.2

./scripts/run_inference.py inference.output_prefix=example_outputs/1N8Zdesign0.2/ inference.input_pdb=examples/input_pdbs/1N8Z-D3_C.pdb 'contigmap.contigs=[C1-121/0 40-80]' 'ppi.hotspot_res=[A11,A19,A34,A45,A75,A76,A79]' inference.num_designs=1200 denoiser.noise_scale_ca=0.2 denoiser.noise_scale_frame=0.2

0.3

./scripts/run_inference.py inference.output_prefix=example_outputs/1N8Zdesign0.3/ inference.input_pdb=examples/input_pdbs/1N8Z-D3_C.pdb 'contigmap.contigs=[C1-121/0 40-80]' 'ppi.hotspot_res=[A11,A19,A34,A45,A75,A76,A79]' inference.num_designs=1200 denoiser.noise_scale_ca=0.3 denoiser.noise_scale_frame=0.3

0.4

./scripts/run_inference.py inference.output_prefix=example_outputs/1N8Zdesign0.4/

inference.input_pdb=examples/input_pdbs/1N8Z-D3_C.pdb 'contigmap.contigs=[C1-121/0 40-80]' 'ppi.hotspot_res=[A11,A19,A34,A45,A75,A76,A79]' inference.num_designs=1200 denoiser.noise_scale_ca=0.4 denoiser.noise_scale_frame=0.4

S4. ProteinMPNN: Sequence Design on Backbones

0

rm check.point 2>/dev/null

../mpnn_fr/dl_interface_design.py -pdbdir inputs/pdbs/1N8Zdesign0 -relax_cycles 2 -seqs_per_struct 10 -outpdbdir ./mpnn_1N8Zdesign0

0.1

rm check.point 2>/dev/null

../mpnn_fr/dl_interface_design.py -pdbdir inputs/pdbs/1N8Zdesign0.1 -relax_cycles 2 -seqs_per_struct 10 -outpdbdir ./mpnn_1N8Zdesign01

0.2

rm check.point 2>/dev/null

../mpnn_fr/dl_interface_design.py -pdbdir inputs/pdbs/1N8Zdesign0.2 -relax_cycles 2 -seqs_per_struct 10 -outpdbdir ./mpnn_1N8Zdesign02

0.3

rm check.point 2>/dev/null

../mpnn_fr/dl_interface_design.py -pdbdir inputs/pdbs/1N8Zdesign0.3 -relax_cycles 2 -seqs_per_struct 10 -outpdbdir ./mpnn_1N8Zdesign03

0.4

rm check.point 2>/dev/null

../mpnn_fr/dl_interface_design.py -pdbdir inputs/pdbs/1N8Zdesign0.4 -relax_cycles 2 -seqs_per_struct 10 -outpdbdir ./mpnn_1N8Zdesign04

S5. Silent_pdb：

0

~/software/silent_tools/silentfrompdbs *_*.pdb > mpnn_1N8Zdesign0.silent

0.1

~/software/silent_tools/silentfrompdbs *_*.pdb > mpnn_1N8Zdesign01.silent

0.2

~/software/silent_tools/silentfrompdbs *_*.pdb > mpnn_1N8Zdesign02.silent

0.3

~/software/silent_tools/silentfrompdbs *_*.pdb > mpnn_1N8Zdesign03.silent

0.4

~/software/silent_tools/silentfrompdbs *_*.pdb > mpnn_1N8Zdesign04.silent

S6. Structure Prediction & Ranking (AlphaFold)

0

rm check.point 2>/dev/null

../af2_initial_guess/predict.py -silent ./mpnn_1N8Zdesign0.silent -outsilent ./af2_1N8Zdesign0_2/af2_1N8Zdesign0.silent

0.1

rm check.point 2>/dev/null

../af2_initial_guess/predict.py -silent ./mpnn_1N8Zdesign01.silent -outsilent ./af2_1N8Zdesign01_2/af2_1N8Zdesign01.silent

0.2

rm check.point 2>/dev/null

../af2_initial_guess/predict.py -silent ./mpnn_1N8Zdesign02.silent -outsilent ./af2_1N8Zdesign02_2/af2_1N8Zdesign02.silent

0.3

rm check.point 2>/dev/null

../af2_initial_guess/predict.py -silent ./mpnn_1N8Zdesign03.silent -outsilent ./af2_1N8Zdesign03_2/af2_1N8Zdesign03.silent

0.4

rm check.point 2>/dev/null

../af2_initial_guess/predict.py -silent ./mpnn_1N8Zdesign04.silent -outsilent ./af2_1N8Zdesign04_2/af2_1N8Zdesign04.silent
